# Positive and Negative Symptoms Changes in Schizophrenia Patients on Antipsychotic Treatment: a Systematic Review and Dose–Response Meta-analysis

**DOI:** 10.1093/schbul/sbag077

**Published:** 2026-08-29

**Authors:** Xiao Lin, Spyridon Siafis, Jing Tian, Hui Wu, Christoph U Correll, Johannes Schneider-Thoma, Stefan Leucht

**Affiliations:** Department of Psychiatry and Psychotherapy, Technical University of Munich, School of Medicine and Health, Klinikum Rechts der Isar, Munich, Bavaria 81675, Germany; German Center for Mental Health (DZPG), partner site Munich/Augsburg, FKZ TUM 01EE2303B 2023-2025, Germany; Department of Psychiatry and Psychotherapy, Technical University of Munich, School of Medicine and Health, Klinikum Rechts der Isar, Munich, Bavaria 81675, Germany; German Center for Mental Health (DZPG), partner site Munich/Augsburg, FKZ TUM 01EE2303B 2023-2025, Germany; Department of Psychiatry and Psychotherapy, Technical University of Munich, School of Medicine and Health, Klinikum Rechts der Isar, Munich, Bavaria 81675, Germany; German Center for Mental Health (DZPG), partner site Munich/Augsburg, FKZ TUM 01EE2303B 2023-2025, Germany; Department of Psychiatry and Psychotherapy, Technical University of Munich, School of Medicine and Health, Klinikum Rechts der Isar, Munich, Bavaria 81675, Germany; German Center for Mental Health (DZPG), partner site Munich/Augsburg, FKZ TUM 01EE2303B 2023-2025, Germany; Department of Psychiatry, Zucker Hillside Hospital, Glen Oaks, New York 11004, United States; Department of Psychiatry and Molecular Medicine, Donald and Barbara Zucker School of Medicine at Hofstra/Northwell, Hempstead, New York 11549, United States; Department of Child and Adolescent Psychiatry, Charité-Universitätsmedizin Berlin, Berlin, Berlin 13353, Germany; German Center for Mental Health (DZPG), partner site Berlin, Germany; Department of Psychiatry and Psychotherapy, Technical University of Munich, School of Medicine and Health, Klinikum Rechts der Isar, Munich, Bavaria 81675, Germany; German Center for Mental Health (DZPG), partner site Munich/Augsburg, FKZ TUM 01EE2303B 2023-2025, Germany; Department of Psychiatry and Psychotherapy, Technical University of Munich, School of Medicine and Health, Klinikum Rechts der Isar, Munich, Bavaria 81675, Germany; German Center for Mental Health (DZPG), partner site Munich/Augsburg, FKZ TUM 01EE2303B 2023-2025, Germany

**Keywords:** clinical efficacy, symptom improvement, psychiatric disorders

## Abstract

**Background and Hypothesis:**

Positive and negative symptoms are core features of schizophrenia and key indicators of antipsychotic efficacy. We aimed to explore the relationship between antipsychotic doses and changes in positive/negative symptoms.

**Study Design:**

We reviewed the Cochrane Schizophrenia Group register (updated on July-26-2024) and prior systematic reviews to identify fixed-dose randomized controlled trials that assessed the monotherapy effects of 21 antipsychotic drugs in acute schizophrenia. The primary outcome measure was the average change in positive and negative symptoms from the studies’ baseline to endpoint, calculated using standard mean differences as the effect size metric. Dose–response relationships were analyzed using random-effects meta-analyses with restricted cubic splines.

**Study Results:**

Finally, a total of 165 studies were identified. 112 studies with 385 dose arms (37 096 participants) reported positive or negative symptoms and were evaluated in meta-analyses. Most antipsychotics showed parallel dose–response curves for positive and negative symptoms, with a smaller magnitude of effect on negative symptoms—except a few drugs, in particular partial dopamine agonists which had at least equal effect sizes for positive and negative symptoms. In most cases both curves declined until reaching an inflection point, then plateaued or efficacy decreased.

**Conclusions:**

The effects of antipsychotics on negative symptoms are smaller than those on positive symptoms, at least in studies of mostly acutely exacerbated patients. Nevertheless, the dose–response curves for both types of symptoms are relatively parallel. This information can assist clinicians in dosing antipsychotics and has implications for the understanding of the antipsychotic mechanism.

## Introduction

Schizophrenia often is a persistent and debilitating disorder. It is composed of positive (such as hallucinations, delusions, disorganized speech and disorganized behavior), negative (such as affective flattening, alogia, avolition, anhedonia, and social withdrawal), cognitive and less frequent, motor symptoms.^1^

Positive and negative symptoms in schizophrenia could arise from distinct neurobiological mechanisms, which may underlie the differential efficacy of antipsychotics across symptom domains. While dopamine D₂ receptor antagonism or partial agonism—shared by all currently approved antipsychotics except xanomeline–trospium—can effectively reduce positive symptoms, it may worsen negative symptoms by impairing dopaminergic reward pathways.^2^ These differences in receptor mechanisms may also contribute to the heterogeneous dose–response profiles observed among antipsychotics,^3^ underscoring the importance of evaluating efficacy and dosing separately for each symptom dimension.

Nevertheless, at first glance, most antipsychotics appear to be effective for both positive and negative symptoms—for instance, as reported in Huhn et al. 2019^4^ and Schneider-Thoma et al. 2026.^5^ This apparent overlap in studies of acutely exacerbated patients with schizophrenia with prominent or predominant positive symptoms, whereby positive symptom improvement can lead to secondary negative symptom improvement,^2^ may obscure underlying differences in how these drugs act on distinct symptom domains, including their dose–response patterns, which warrants further investigation.

To address these clinically relevant questions, we conducted dose–response meta-analyses of antipsychotic medications targeting both positive and negative symptoms in diverse patient populations.

## Methods

### Search Strategy

The PRISMA reporting guideline (Supplementary Appendix 1: checklist) was followed, and a protocol was pre-registered at PROSPERO (registration number: CRD42020181467).^6^ Differences between the review and the protocol are outlined in Supplementary Appendix 2.

We conducted a search of the Cochrane Schizophrenia Group’s (CSzG’s) Study-Based Register up to July 26, 2024. Further details on the search strategy and a PRISMA-flow diagram can be found in Supplementary Appendices 3 and 4.

### Selection Criteria

#### Participants

We included participants diagnosed with schizophrenia or related disorders based on criteria such as the International Classification of Diseases, Diagnostic and Statistical Manual of Mental Disorders, or clinical diagnosis. Participants with stable schizophrenia were excluded due to methodological and clinical heterogeneity. Data were analyzed separately for the following population subgroups: (1) working-age adults (18–65 years old) with multi-episode schizophrenia and acute exacerbations (including patients with treatment-resistant schizophrenia because there is no clear evidence that antipsychotic dose effects differ based on treatment resistance^7^), (2) patients with predominant negative symptoms, defined as patients with more severe negative than positive symptoms and only mild positive symptoms (following the framework operationalized by Krause et al.^8^), (3) patients with first-episode schizophrenia, as defined by the original authors,^9^ (4) elderly patients (>65 years old), and (5) children and adolescents (<18 years old).

#### Interventions

We included 20 second-generation antipsychotics (SGAs), all used as monotherapy: amisulpride, aripiprazole, asenapine, blonanserin, brexpiprazole, cariprazine, clozapine, haloperidol, iloperidone, lumateperone, lurasidone, olanzapine, olanzapine-samidorphan, paliperidone, perospirone, quetiapine, risperidone, sertindole, xanomeline /xanomeline-trospium, ziprasidone, and zotepine. This selection comprises all SGAs currently available in Europe and/or the United States. The list of eligible agents was prespecified in the study protocol and is consistent with our previous publication.^6^^,^^10–13^ All formulations of these SGAs were eligible. Haloperidol was included as it is frequently used as an active comparator in monotherapy trials. The choice of drugs was also based on previous meta-analyses (in particular Huhn et al.^4^), which showed that fixed-dose, dose-finding studies for other older drugs are largely unavailable.

#### Comparator

Comparators were different doses of the same antipsychotics and placebo.

#### Outcome

The two co-primary outcomes were the standardized mean difference (SMD) of the mean change from baseline to endpoint or the endpoint for positive/negative symptoms. The scales we used at least once for positive and negative symptoms are listed in Supplementary Appendix 5.

#### Study Design

Randomized controlled trials (RCTs), either blinded or open-labelled, using fixed or narrowly defined dose ranges were included. Relapse prevention studies (studies in stable schizophrenia) were excluded.

Further details on the selection criteria can be found in our a priori published protocol and in our previous publications.^6^^,^^10–13^

### Data Extraction, Assessment, and Analysis

Two reviewers (among SL, SS, HW, XL, or JT) independently screened the references, extracted the data, and evaluated the risk of bias using the Cochrane Risk of Bias Tool 1.^14^ Disagreements were resolved by consensus.

A one-stage dose–response analysis was performed within a frequentist framework using restricted cubic splines, following the method developed by Crippa and Orsini.^15^^,^^16^ Knots were placed at the 25^th^, 50^th^, 75^th^ percentiles, except for asenapine, where knots were positioned at the 10^th^, 50^th^, 90^th^ percentiles, consistent with prior studies.^10–12^ For drugs with sufficient data, we applied a Wald chi-squared test for the splines coefficients to evaluate the overall dose–response relationship.^16^ Corresponding *P*-values were reported. A two-sided alpha level of 0.05 was used to determine statistical significance. Different formulations of the same drug were combined by converting them into their equivalent oral doses (refer to Supplementary Appendix 6).

To assess the robustness of the outcomes, sensitivity analyses were conducted for each patient-group by: (1) excluding studies that compared a single dose of antipsychotics with placebo, (2) removing studies involving treatment-resistant patients, and (3) analyzing different drug formulations separately.

Heterogeneity in the dose–response meta-analysis was quantified using the Variance Partition Coefficient (VPC), a multivariate extension of the I^2^ statistic.^15^ For comparisons involving more than 10 trials, small-study effects and publication bias were examined using funnel plots and Egger’s tests.^14^ Data analysis was performed with the R package dosresmeta,^15^^,^^16^ while the R package meta (v4.12–0)^17^ was employed to evaluate small-study bias.

## Results

We identified 153 studies from previous publications,^10–12^ 164 additional reports from a specific search for blonanserin, perospirone, and samidorphan (not included in our previous dose–response meta-analyses), and 359 reports from searches in the CSzG’s register. After screening, 165 studies met our inclusion criteria (PRISMA diagram in Supplementary Appendix 4). Positive symptom data were available in 110 studies (380 drug arms, N = 36 372 participants), and negative symptom data in 111 studies (380 drug arms, N = 37 006). Most study participants were working-age patients (18–65 years old) experiencing acute exacerbation of chronic schizophrenia. Four studies focused on participants with predominant negative symptoms (all evaluated amisulpride, with one also evaluating olanzapine, N = 729). Three studies focused on participants with a first episode of schizophrenia. Eight studies targeted children or adolescents (N = 1778), while none were conducted exclusively in elderly populations. Detailed descriptions of the included studies are presented in Supplementary Appendix 7 (Tables S2 and S3) and their risk of bias is provided in Supplementary Appendix 8.

**Figure 1:**
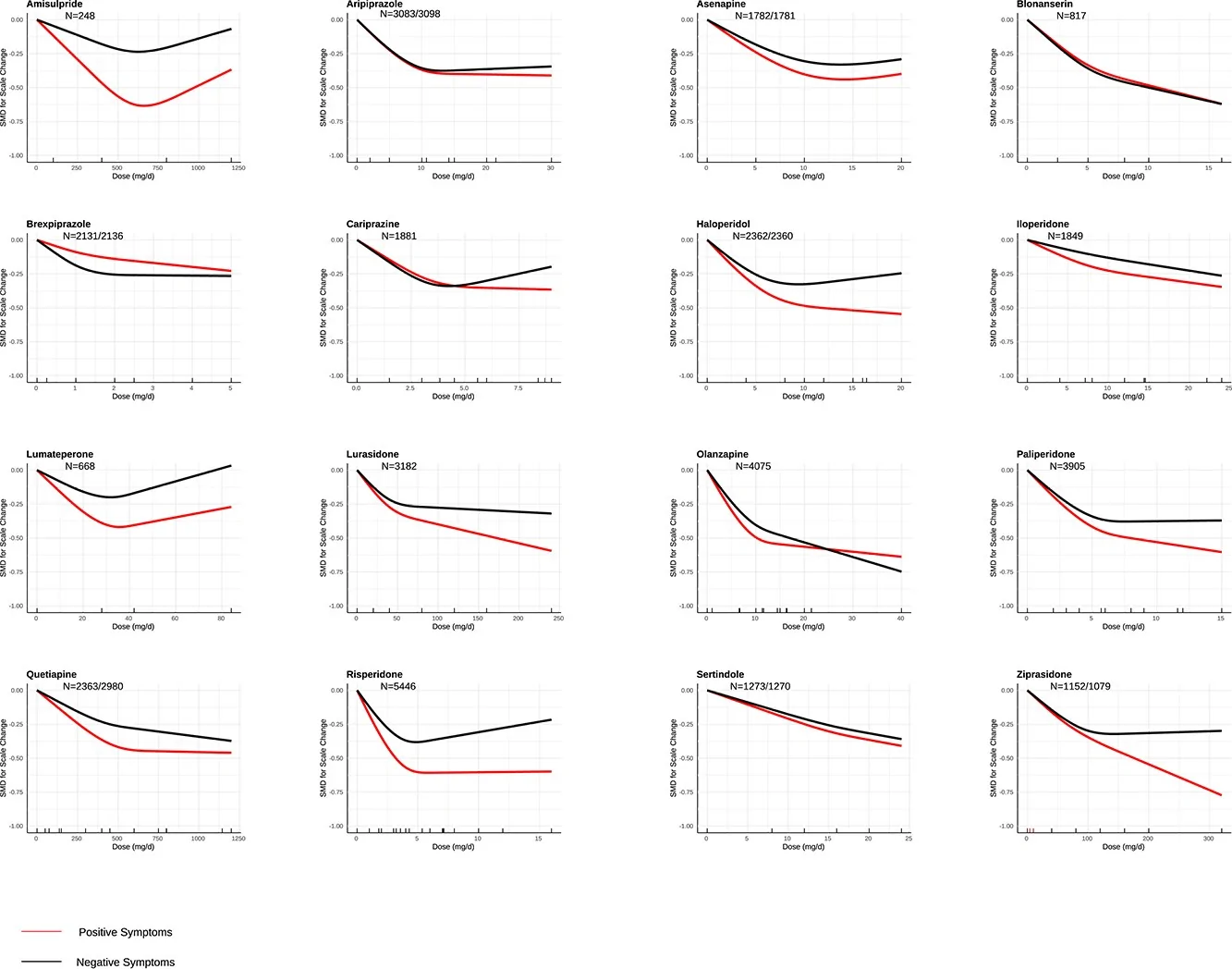
Positive/negative symptoms change of individual antipsychotics in different dosages in adult participants with acute exacerbations of chronic schizophrenia. The dose-response curve depicts the SMD of positive/negative change comparing a given dose of a drug to 0 mg/d or placebo. The knot locations are set at the 25^th^, 50^th^, and 75^th^ percentiles to anchor the curve, except for asenapine, which is set at the 10^th^, 50^th^, and 90^th^ percentiles. Different formulations are pooled in aripiprazole, asenapine, blonanserin, olanzapine, paliperidone, quetiapine, and risperidone studies. N: number of participants (positive symptoms/negative symptoms; if a single number is shown, the sample size is identical for both outcomes).

**Figure 2:**
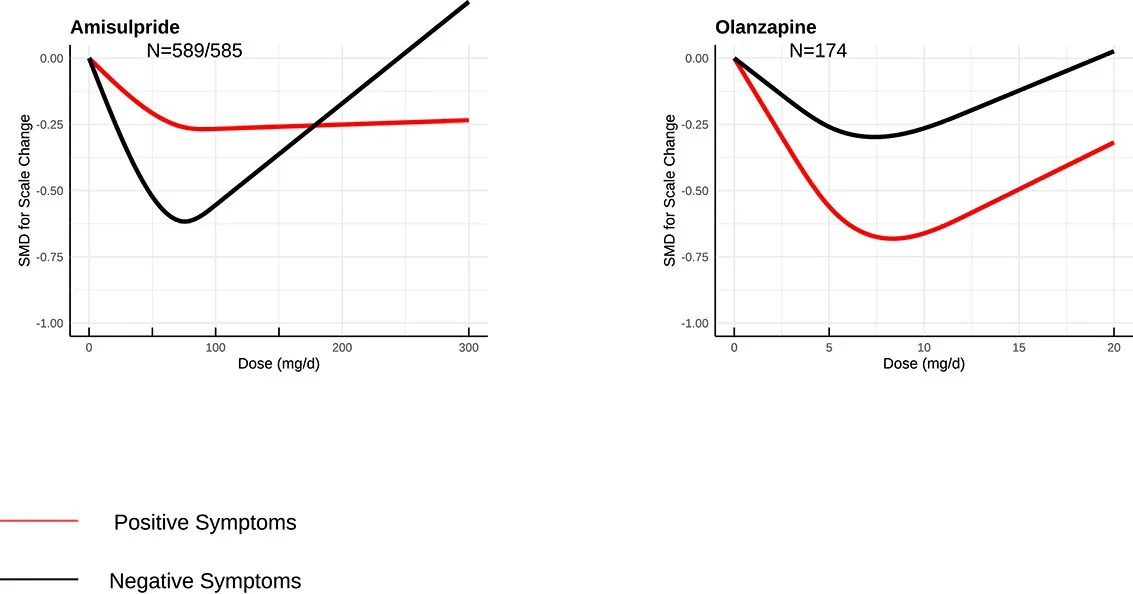
Positive/negative symptoms change of individual antipsychotics in different dosages in participants with predominant negative symptoms of chronic schizophrenia. The dose-response curve depicts the SMD of positive/negative change comparing a given dose of a drug to 0 mg/d or placebo. The knot locations are set at the 25^th^, 50^th^, and 75^th^ percentiles to anchor the curve. N: number of participants (positive symptoms/negative symptoms; if a single number is shown, the sample size is identical for both outcomes).

**Figure 3:**
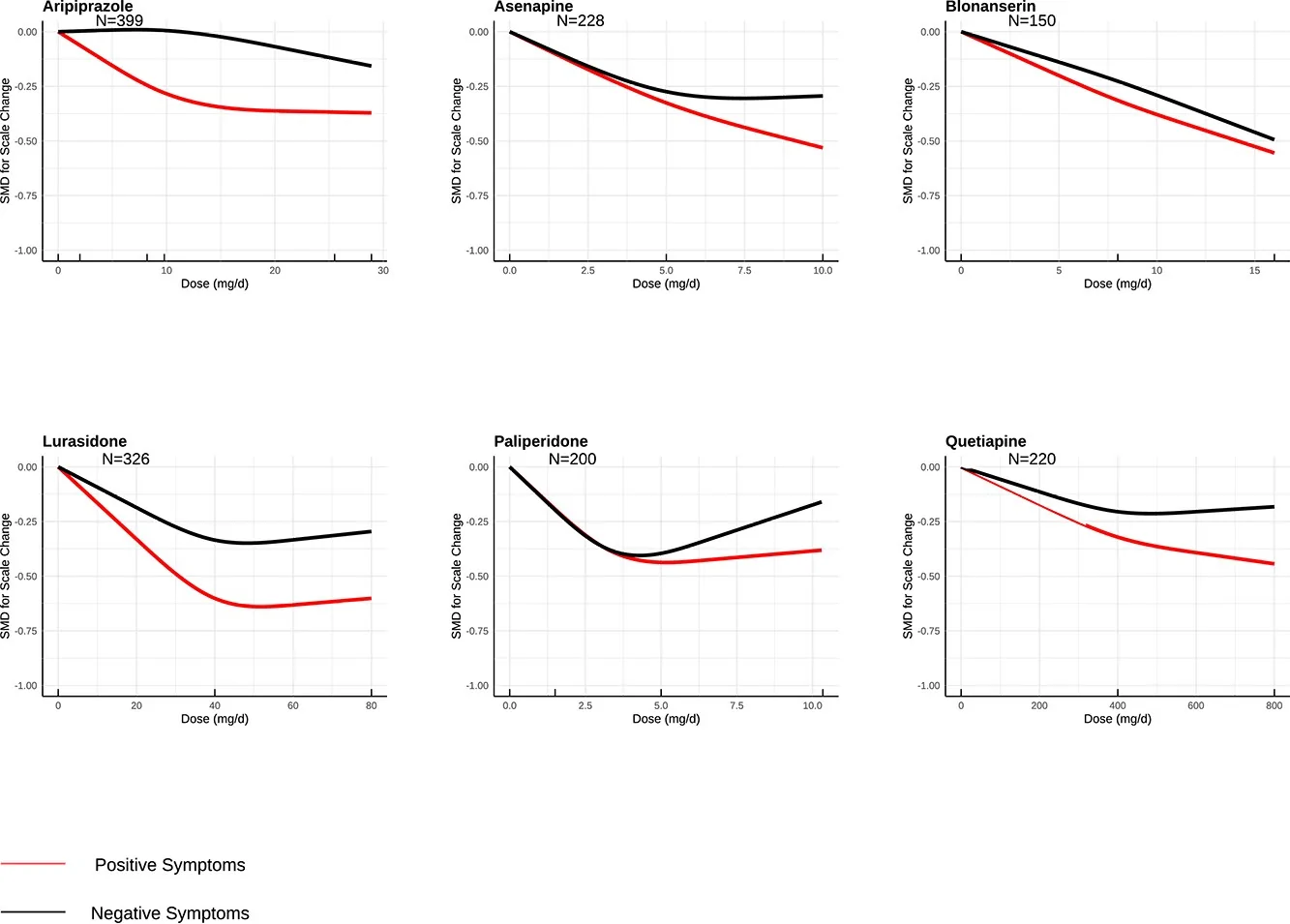
Positive/negative symptoms change of individual antipsychotics in different dosages in children/ adolescent participants with acute exacerbations of chronic schizophrenia. The dose-response curve depicts the SMD of positive/negative change comparing a given dose of a drug to 0 mg/d or placebo. The knot locations are set at the 25^th^, 50^th^, and 75^th^ percentiles to anchor the curve, except for asenapine, which is set at the 10^th^, 50^th^, and 90^th^ percentiles. N: number of participants.

**Figure 4:**
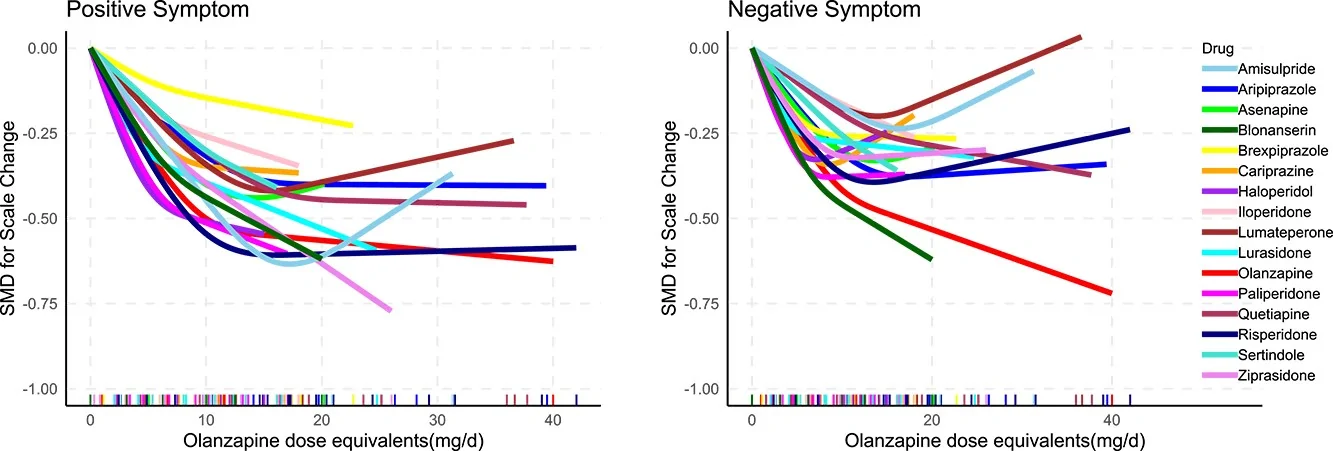
Olanzapine-equivalent dose–response curves for positive/negative symptoms across individual antipsychotics in adults with acute exacerbations of chronic schizophrenia. SMD: standardized mean difference.

**Figure 5:**
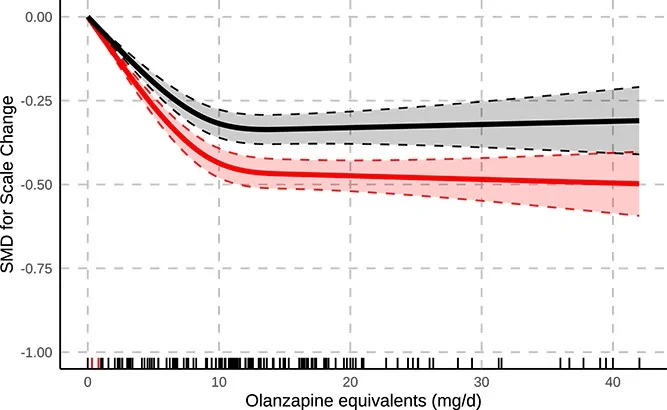
Olanzapine-equivalent dose–response curves for pooled antipsychotics in adults with acute exacerbations of chronic schizophrenia. The upper curve represents negative symptoms, and the lower curve represents positive symptoms. SMD: standardized mean difference.

**Figure 6:**
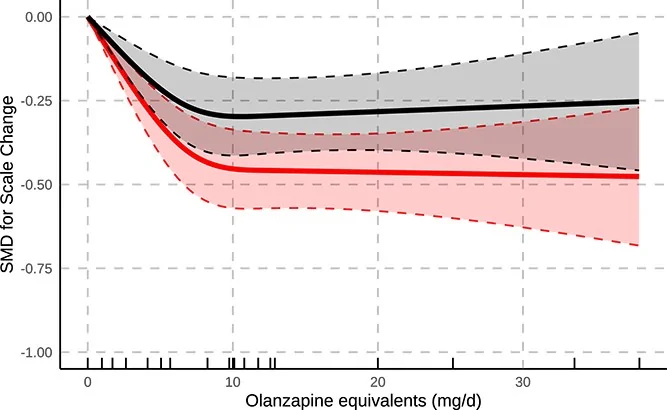
Olanzapine-equivalent dose–response curves for pooled antipsychotics in children/adolescent with acute exacerbations of chronic schizophrenia. The upper curve represents negative symptoms, and the lower curve represents positive symptoms. SMD: standardized mean difference.

Figures 1–6 provide an overview of the dose–response curves: adults with acute exacerbations (Figure 1; curves after conversion to olanzapine-equivalents in Figure 4), patients with predominant negative symptoms (Figure 2), and children and adolescents (Figure 3). Figures 5 and 6 show the dose–response curves after pooling all drugs after conversion to olanzapine equivalents. In the following, we provide an overview of the drugs and the characteristics of their dose–response curves in alphabetical order (detailed information in Supplementary Appendix 9). Model coefficients with 95% confidence intervals (CIs) are also reported in Supplementary Appendix 9, while maximum predicted effects at individual doses are presented in the text without separate CIs. Additional figures with CIs are provided in Supplementary Appendix 10.

### Amisulpride

One oral dose-finding study was included in the group of patients with chronic schizophrenia during an acute exacerbation. It is a special situation because the study^18^ compared 100 mg, 400 mg, 800 mg, and 1200 mg amisulpride per day, thus there was no placebo control.

For both positive and negative symptoms, the curve declined (meaning a reduction of symptoms) until doses up to around 625 mg/d, with the reduction in the positive symptom curve being approximately twice that of the negative symptom (Table 1.1, Figures 1 and 4). After the lowest point, the two curves showed a parallel, gradual increase that remained below 0.

**Table 1:** Summary of Dose–Response Characteristics of Individual Antipsychotics for Positive and Negative Symptoms. **Table 1.1** Adult Participants with Acute Exacerbations of Chronic Schizophrenia.

Drug	Outcome	N (studies)	N (arms)	N (participants)	Dose range examined(mg/d) ^a^	Approximate plateau Dose (mg/d) ^b^	Minimum SMD (Maximum efficacy)	*P*-value	Shape of curve
Amisulpride	PS	1	4	248	100-1200	660.8	−0.63	.0262^*^	bell-shaped
	NS	1	4	248	100-1200	625.6	−0.24	.5552	bell-shaped
Aripiprazole	PS	11	31	3083	2-30	12	−0.41	.0000^*^	plateau
	NS	11	31	3098	2-30	12	−0.38	.0000^*^	plateau
Asenapine	PS	5	14	1782	5-20	14	−0.44	.0000^*^	plateau
	NS	5	14	1781	5-20	14	−0.33	.0002^*^	plateau
Blonanserin	PS	2	7	817	2.5-16	/ ^c^	−0.62	.0000^*^	monotonous
	NS	2	7	817	2.5-16	/	−0.62	.0008^*^	monotonous
Brexpiprazole	PS	4	17	2131	.25-5	/	−0.23	.0116^*^	monotonous
	NS	4	17	2136	.25-5	2	−0.27	.0000^*^	plateau
Cariprazine	PS	5	16	1881	1.5-9	4	−0.37	.0000^*^	plateau
	NS	5	16	1881	1.5-9	4.3	−0.34	.0000^*^	bell-shaped
Haloperidol	PS	15	32	2362	4-20	10	−0.55	.0000^*^	plateau
	NS	15	32	2360	4-20	9.4	−0.33	.0000^*^	bell-shaped
Iloperidone	PS	4	12	1849	4-24	/	−0.35	.0000^*^	monotonous
	NS	4	12	1849	4-24	/	−0.26	.0005^*^	monotonous
Lumateperone	PS	2	6	668	28-84	35.6	−0.42	.0017^*^	bell-shaped
	NS	2	6	668	28-84	31.3	−0.19	.1143	bell-shaped
Lurasidone	PS	10	29	3182	20-240	/	−0.59	.0000^*^	monotonous
	NS	10	29	3182	20-240	50	−0.32	.0000^*^	plateau
Olanzapine	PS	19	46	4075	1-40	12	−0.64	.0000^*^	plateau
	NS	19	46	4075	1-40	/	−0.75	.0000^*^	monotonous
Paliperidone	PS	10	33	3905	2-15	6	−0.60	.0000^*^	plateau
	NS	10	33	3905	2-15	6	−0.38	.0000^*^	plateau
Quetiapine	PS	8	30	2363	75-1200	600	−0.46	.0084^*^	plateau
	NS	9	33	2980	50-1200	500	−0.37	.0054^*^	plateau
Risperidone	PS	20	54	5446	1-16	5	−0.61	.0000^*^	plateau
	NS	20	54	5446	1-16	4.8	−0.38	.0000^*^	bell-shaped
Sertindole	PS	4	15	1273	8-24	/	−0.41	.0000^*^	monotonous
	NS	4	15	1270	8-24	/	−0.36	.0001^*^	monotonous
Ziprasidone	PS	6	18	1152	4-320	/	−0.77	.0000^*^	monotonous
	NS	5	14	1079	40-320	120	−0.32	.0000^*^	plateau

**Table 1.2:** Participants with Predominant Negative Symptoms.

Drug	Outcome	N (studies)	N (arms)	N (participants)	Dose range examined (mg/d) ^a^	Approximate plateau Dose (mg/d) ^b^	Minimum SMD (Maximum efficacy)	*P*-value	Shape of curve
Amisulpride	PS	4	10	589	50-300	75	−0.27	.2288	plateau
	NS	4	10	585	50-300	75.6	−0.62	.0000^*^	bell-shaped
Olanzapine	PS	1	3	174	5-20	8.3	−0.68	0.0276^*^	bell-shaped
	NS	1	3	174	5-20	7.4	−0.30	0.2024	bell-shaped

**Table 1.3:** Children/Adolescent Participants.

Drug	Outcome	N (studies)	N (arms)	N (participants)	Dose range examined (mg/d) ^a^	Approximate plateau Dose (mg/d) ^b^	Minimum SMD (Maximum efficacy)	*P*-value	Shape of curve
Aripiprazole	PS	2	6	399	2-30	17.5	−0.37	.0114^*^	plateau
	NS	2	6	399	2-30	/^c^	−0.16	.3992	monotonous
Asenapine	PS	1	3	228	5-10	/	−0.53	.0043^*^	monotonous
	NS	1	3	228	5-10	6	−0.30	.1283	plateau
Blonanserin	PS	1	3	150	8-16	/	−0.55	.0244^*^	monotonous
	NS	1	3	150	8-16	/	−0.49	.0513	monotonous
Lurisidone	PS	1	3	326	40-80	50	−0.64	.0000^*^	plateau
	NS	1	3	326	40-80	50	−0.35	.0258^*^	plateau
Paliperidone	PS	1	4	200	1.5-12	5	−0.44	.0562	plateau
	NS	1	4	200	1.5-12	4.3	−0.41	.1438	bell-shaped
Quetiapine	PS	1	3	220	400-800	400	−0.44	.0227^*^	plateau
	NS	1	3	220	400-800	400	−0.21	.3985	plateau

a: Oral equivalence dose; b:The column “Approximate plateau dose” also includes the dose with the highest efficacy in bell-shaped curves; c: For drugs with a monotonous dose–response shape, no clear dose for near-maximum efficacy can be identified, in such cases, “/” is used. *P < .05, doses have a significant effect on the efficacy. N = number; SMD = standardized mean difference; PS = positive symptoms; NS = negative symptoms.

Four oral studies were included in the group of patients with predominant negative symptoms, including two placebo-controlled single-dose studies and two dose-finding studies.

For positive symptoms, the curve declined at doses up to around 75 mg/d, after which it remained essentially flat (Figure 2 and Table 1.2). For negative symptoms, the curve declined sharply at doses up to around 75 mg/d, after which it rose rapidly, indicating reduced efficacy (Figure 2 and Table 1.2).

### Aripiprazole

Eleven studies were included in the adult group investigating oral aripiprazole (nine studies), aripiprazole lauroxil (one study)^19^, and aripiprazole monohydrate (one study).^20^ Among them, five single-dose studies with a placebo control and six dose-finding studies were included.

The two dose–response curves were nearly overlapping, not only in shape but also in effect sizes meaning that the efficacy for positive and negative symptoms was the same. Both curves showed a decline at the beginning before reaching a low point at approximately 12 mg/d, after which they plateaued (Table 1.1, Figures 1 and 4).

Two dose-finding studies were included in the child/adolescent group investigating oral aripiprazole.

The curves are different from those in adults: The curve for positive symptoms declined first then reached a plateau at approximately 17.5 mg/d (Figure 3, Table 1.3). In contrast, the negative symptoms curve decreased only slightly, ultimately ending at a higher level than that of the positive symptoms curve, suggesting a small and less pronounced improvement in negative symptoms (Figure 3, Table 1.3).

### Asenapine

Four oral and one transdermal study were included in adult group investigating asenapine. Except for one oral study, all others were dose-finding studies.

The two dose–response curves showed an initial decline, reaching a low point at approximately 14 mg/d, after which they plateaued in parallel. In the end, the negative symptom curve was somewhat higher (Table 1.1, Figures 1 and 4).

One dose-finding study was included in the child/adolescent group investigating oral asenapine.

The positive symptom curve showed a monotonous decrease, while the curve for negative symptoms was declining first then reached a plateau at approximately 6 mg/d (Figure 3, Table 1.3).

### Blonanserin

One oral and one transdermal study were included in adult group investigating blonanserin. They were all dose-finding studies.

The two dose–response curves nearly overlapped, that is, suggesting the same effect sizes and showing an initially sharp decline that became more gradual after about 6 mg/d (Table 1.1, Figures 1 and 4).

One dose-finding study was included in the child/adolescent group investigating oral blonanserin.

Both the positive and negative symptom curves showed a monotonous decrease, with the negative symptom curve being only slightly higher (Figure 3, Table 1.3).

### Brexpiprazole

Four oral dose-finding studies were included.

The positive symptom curve showed a monotonous decrease. The curve for negative symptoms declined first then reached a plateau at approximately 2 mg/d, with an ending a little bit lower than positive symptoms (Table 1.1, Figures 1 and 4). In contrast to most other drugs (except amisulpride for patients in predominant negative symptoms), the maximum effect size for negative symptoms was somewhat higher than that for positive symptoms.

### Cariprazine

Five oral dose-finding studies were included.

The two dose–response curves were nearly overlapping, meaning very similar effect sizes, and initially declining before reaching a low point at approximately 4 mg/d. Subsequently, the positive symptom curve plateaued, while the negative symptom curve increased meaning less efficacy (Table 1.1, Figures 1 and 4).

### Clozapine

No fixed-dose study reported changes in positive/negative symptoms.

### Haloperidol

Fifteen studies were included in investigating oral haloperidol. Fourteen placebo-controlled single-dose studies and one dose-finding studies were included.

The two dose–response curves initially declined to a low point at approximately 10 mg/d for positive symptoms and 9 mg/d for negative symptoms. After this, the positive symptom curve plateaued, while the negative symptom curve increased, indicating reduced efficacy at higher doses. In the end, the negative symptom curve was higher, meaning less efficacy (Table 1.1, Figures 1 and 4).

Because of insufficient data, we could not build a dose–response curve for the study in first episode patients in the dataset^21^ which investigated haloperidol.

### Iloperidone

Four studies were included in investigating oral iloperidone. One placebo-controlled and three dose-finding studies were included.

The two dose–response curves both showed a monotonous decrease. In the later stages, the two curves were nearly parallel, with the negative symptom curve positioned higher (Table 1.1, Figures 1 and 4).

### Lumateperone

Two dose-finding oral studies were included.

The dose–response curves were both bell-shaped. A low point was reached at approximately 35 mg/d. The negative symptom curve consistently positioned higher (Table 1.1, Figures 1 and 4).

### Lurasidone

Ten oral studies were included in the adult group investigating lurasidone. Eight of them were dose-finding studies.

The two dose–response curves initially declined to a low point at approximately 50 mg/d, where was a knot. After this, the negative symptom curve plateaued, while the positive symptom curve continued to decline at a slightly more gradual rate (Table 1.1, Figures 1 and 4).

One dose-finding study was included in the child/adolescent group investigating oral lurasidone.

The two dose–response curves showed a decline at the beginning before reaching a low point again at approximately 50 mg/d, after which they plateaued. As for most drugs, the negative symptom curve was higher (Figure 3, Table 1.3).

### Olanzapine

Fourteen placebo-controlled and five dose-finding studies (one of them being a long-acting injectable [LAI] study^22^) were investigated.

The dose–response curves initially decreased, with the negative symptom curve being slightly higher. After a turning point at approximately 12 mg/d, the rate of decline for both curves slowed, but the decline of the positive symptom curve slowed more clearly. The two curves intersected at approximately 25 mg/d. At the end, the positive symptom curve was slightly higher (Table 1.1, Figures 1 and 4).

One dose-finding oral study was included in the group of patients with predominant negative symptoms.

The dose–response curves were both bell-shaped and had a low point at approximately 8 mg/d, and the negative symptom curve positioned above the positive symptom curve, indicating lower efficacy (Figure 2, Table 1.2).

### Olanzapine-Samidorphan

No fixed-dose study reported changes in positive/negative symptoms.

### Paliperidone

Ten studies, five LAI and five oral were included in the adult group. Eight of them were dose-finding studies.

The two dose–response curves initially declined to a low point at approximately 6 mg/d. After this, the negative symptom curve plateaued, while the positive symptom curve continued to decline at a slightly more gradual rate (Table 1.1, Figures 1 and 4).

One dose-finding study was included in the child/adolescent group investigating oral paliperidone.

The two dose–response curves initially nearly overlapped until they declined to a low point. Following this, the positive symptom curve plateaued at approximately 5 mg/d, while the negative symptom curve, in contrast, began to increase at approximately 4 mg/d (Figure 3, Table 1.3).

### Perospirone

No fixed-dose study reported changes in positive/negative symptoms.

### Quetiapine

Eight studies for positive symptoms and nine for negative symptoms were included in adult group investigating oral quetiapine. Two placebo-controlled and six/seven (positive/negative symptom) dose-finding studies were included.

The two dose–response curves initially showed a decline, reaching a low point at approximately 500 mg/d (negative symptoms) and 600 mg/d (positive symptoms). After this, the positive symptom declined only gradually, while the negative symptom curve continued to decline at a somewhat greater rate. At the end, the negative symptom curve was still slightly higher (Table 1.1, Figures 1 and 4).

One dose-finding studies was included in the child/adolescent group investigating oral quetiapine.

The two dose–response curves initially showed a decline, reaching a low point for negative symptoms at approximately 400 mg/d. After this, the negative symptom curve plateaued, while the positive symptom curve continued to decline at a more gradual rate. At the end, the negative symptom curve was higher (Figure 3, Table 1.3).

Due to insufficient data, we could not build a dose–response curve for the only study that investigated quetiapine in first-episode patients.

### Risperidone

Twenty studies in the adult group were investigated, from which eleven were single-dose studies with a placebo control and nine were dose-finding studies. Concerning formulation, four were LAIs (two risperidone microspheres,^23^^,^^24^ one risperidone ISM, a new intramuscular injection,^25^ and one risperidone RBP-7000, a sustained-release subcutaneous injection^26^), and 16 were oral studies.

The two dose–response curves initially declined to a low point at approximately 5 mg/d (with the negative symptom curve being higher). Afterward, the positive symptom curve plateaued, while the negative symptom curve, in contrast, began to rise meaning less efficacy. Ultimately, the negative symptom curve was higher (Table 1.1, Figures 1 and 4).

Due to insufficient data, we could not build a dose–response curve for the only study that investigated risperidone in the child/adolescent group and the first-episode patient group separately.

### Sertindole

Four dose-finding oral studies were included.

The two dose–response curves both showed a monotonic decrease. The dose–response curves almost overlapped, with the negative symptom curve being slightly higher (Table 1.1, Figures 1 and 4).

### Xanomeline/Xanomeline-Trospium

There was no fixed-dose study.

### Ziprasidone

There were six/five (positive/negative) oral formulation studies, one was placebo-controlled and five/four (positive/negative) dose-finding studies.

The two dose–response curves initially nearly overlapped as they declined to a low point of the negative symptom curve at approximately 120 mg/d. Following this, the negative symptom curve plateaued, while the positive symptom curve continued to decrease (Table 1.1, Figures 1 and 4).

### Zotepine

There is one study that reported negative symptom change. However, we were unable to build a dose–response curve from the only placebo-controlled single-dose study identified.

### Olanzapine-Equivalent Curves Pooling All Drugs for Adults with Acute Exacerbations

95 studies for positive symptoms (N = 33 163 participants) and 95 for negative symptoms (N = 33 720 participants, the only study for zotepine was not applicable) were included in the dose–response analysis pooling studies in adults with acute exacerbations across drugs. Olanzapine-equivalent doses in the individual arms ranged from 0.3-42.0 mg/d.

The two dose–response curves showed a parallel course and declined at the beginning before reaching a low point at approximately 12 mg/d, after which they plateaued (Figure 5). The curve for negative symptoms plateaued at a higher level, meaning that the effect size for negative symptoms was smaller than that for positive symptoms (positive symptoms: maximum SMD -0.50 at 42.0 mg/d, Wald test, *P* < .01; negative symptoms: maximum SMD -0.34 at 13.6 mg/d, *P* < .01).

### Olanzapine-Equivalent Curves Pooling all Drugs for Children/Adolescents with Acute Exacerbations

Eight studies (N = 1778 participants) were included in the dose–response analysis pooling studies in children/adolescents with acute exacerbations across drugs. Olanzapine-equivalent doses in the individual arms ranged from 1.0-38.0 mg/d.

The overall pattern was the same as that in adults. Both dose–response curves were parallel. They showed a decline at the beginning before reaching a low point at approximately 9 mg/d, after which they plateaued (Figure 6). The curve for negative symptoms plateaued at a higher level meaning that the effect size for negative symptoms was smaller than that for positive symptoms (positive symptoms: maximum SMD -0.48 at 38.0 mg/d; negative symptoms, maximum SMD -0.30 at 10.6 mg/d; for both curves: *P* < .01).

### Sensitivity Analyses

Most results remained robust in the sensitivity analyses (detailed explanation in Supplementary Appendix 11).

Notably, when only “true” dose-finding studies were analyzed (i.e., excluding the studies which compared only one fixed-dose with placebo), the most important difference was that haloperidol demonstrated a bell-shaped dose–response curve shifted to the left compared to the main analysis, low points at approximately 7.5 mg/d for positive symptoms and 6 mg/d for negative symptoms and diminishing efficacy at higher doses (Supplementary Figure S5). Under the same conditions, amisulpride in patients with predominant negative symptoms showed both the negative symptoms and the positive symptom curve plateauing at approximately 75 mg/d (Supplementary Figure S5). For quetiapine, the dose–response curve of the extended-release formulation was lower than that of the immediate release formulation (Supplementary Figure S7).

### Heterogeneity Assessments

No substantial heterogeneity (i.e., median VPC < 50%) was observed for most antipsychotics. Exceptions included all analyses of amisulpride, blonanserin, lumateperone, and quetiapine, as well as all subgroups with predominant negative symptoms and those involving children or adolescents (detailed in Supplementary Appendix 12).

### Small-Study Effect/Publication Bias

No indications for small-study effects or publication bias were found (detailed in Supplementary Appendix 13).

## Discussion

This meta-analysis examined the dose–response relationships of 20 SGAs and haloperidol across various patient groups, including those with acute exacerbations, predominant negative symptoms, and children/adolescents. Compared to a previous study by Sabe et al., which excluded trials which compared single-doses with placebo trials,^27^ our expanded inclusion criteria added 72 more trials, potentially improving the robustness and accuracy of the observed dose–response estimates.

There are several general patterns: First, for all antipsychotics, both positive and negative symptoms improved with increasing doses at least to some point. Second, positive and negative symptom curves typically followed parallel patterns—at least before the inflection points (“knot points”) of the dose–response curves. Third, these patterns were not only found in adult patients but in children/adolescents, as well. Fourth, improvements were generally greater for positive symptoms, consistent with previous findings.^4^^,^^5^^,^^28^^,^^29^

The main exceptions from the fourth pattern were the partial dopamine agonists aripiprazole, brexpiprazole, and cariprazine. These drugs showed similar reductions in positive and negative symptoms, and for brexpiprazole the pattern was even reversed in that it reduced negative symptoms more than positive symptoms. This pattern may reflect the mechanism of action of partial dopamine receptor agonists which, at least in theory, should reduce dopamine transmission where it is too much (positive symptoms), and enhance it where it is not enough (negative symptoms). Moreover, it should be noted that some observed improvements in negative symptoms may reflect amelioration of secondary negative symptoms—such as those associated with positive symptoms or depression,^2^ rather than direct effects on primary negative symptoms.

Some dose–response curves continued to decline (better efficacy) even at the highest doses examined (in particular blonanserin, iloperidone, sertindole, and the positive symptom curves for brexpiprazole, lurasidone, and ziprasidone, as well as the negative symptom curve for olanzapine). This could mean that the most efficacious doses of these drugs have not been identified yet, making further dose–response studies warranted. Sometimes, however, such attempts are limited by toxicity found in animal studies.

Other dose–response relationships followed a bell-shaped pattern meaning that efficacy first increased with dose but decreased after the knot point. This pattern was observed for amisulpride and lumateperone, and for negative symptoms with cariprazine, haloperidol, and risperidone. Additionally, in patients with predominant negative symptoms, the same pattern was observed for negative symptoms with amisulpride. For negative symptoms such curves could in part be explained by too strong dopamine D_2_ receptor blockade leading to reduced motivation, or to emotional blunting as a consequence of extrapyramidal side-effects at high doses. Indeed, the relatively strong dopamine antagonists amisulpride, haloperidol and risperidone showed this pattern of diminished improvement in negative symptoms at higher doses, potentially reflecting the emergence of negative symptoms secondary to strong dopamine blockade. In contrast, some compounds with broader receptor activity including additional serotonergic mechanism (such as olanzapine or sertindole), may be less prone to this effect.

Most curves, however, plateaued at moderate to high doses, and this pattern also came out in the dose–response curve across all drugs in Figure 5 (sensitivity analysis excluding haloperidol is presented in Supplementary Appendix 11, Figure S8). It may reflect the pharmacodynamic saturation of the drugs’ primary mechanism of action, i.e., blockade or partial agonism of dopamine D_2_ receptors. Once this mechanism of action is saturated, no gain in efficacy can be expected. The therapeutic window for dopamine blockade is approximately 65% to 80%.^10^^,^^30^ Further dose escalation beyond this range has been shown to lead to more side-effects of several drugs.^10–13^

Dose–response analyses for patients with predominant/persistent/primary negative symptoms were only possible for amisulpride and olanzapine, and the evidence for the efficacy of antipsychotics in this patient group is generally limited. In the most comprehensive meta-analysis on this topic to date only low-dose amisulpride (50-300 mg/d where amisulpride is thought to be a dopamine enhancer rather than blocker)^31^ and cariprazine (a stronger D_3_ rather than D_2_ partial agonist)^32^ had reasonable evidence for efficacy on primary negative symptoms. In our study, dose-finding studies for cariprazine in participants with persistent negative symptoms were not available. In four studies on low-dose amisulpride in patients with predominant negative symptoms, the optimal dose for negative symptoms was approximately 75 mg/d, while higher doses appeared less effective—or even harmful—again possibly due to excessive dopamine blockade affecting the reward system (Figure 2). However, this effect diminished after excluding two single-dose studies (see Supplementary Appendix 11). The other compound with data was olanzapine, where in a single trial 5 mg/d was more effective than placebo and 20 mg/d (Lecrubier et al.^33^). Interestingly, even in this subgroup with by definition few positive symptoms, olanzapine reduced positive symptoms more than negative symptoms (Figure 2).

In children and adolescents both the maximum effect sizes (also see Pagsberg et al.^34^) and the shapes of the dose–response curves generally resembled those seen in adults with acute exacerbations, particularly at low to moderate SGA doses. This result is particularly evident when the evidence of all 8 studies with 1778 participants were evaluated in one dose–response meta- analysis, see Figure 6. It is important to know for clinicians that overall the same dose–response relationships can be expected in this subgroup, but more data for individual drugs are needed. Data for elderly patients are lacking, and available data for first-episode cases are too sparse for meaningful analysis.

In the sensitivity analysis including only true dose-finding studies (i.e., excluding studies compared a single dose with placebo), the dose–response curve for haloperidol shifted to the left and showed a bell-shaped pattern. In the primary analysis, efficacy for positive symptoms started to plateau at approximately 10 mg/d, whereas for negative symptoms it reached a minimum at around 9 mg/d and then increased, indicating reduced efficacy at higher doses. When only true dose-finding studies were included, optimal efficacy was observed at approximately 7.5 mg/d for positive symptoms and 6 mg/d for negative symptoms, respectively. This may be because the excluded studies almost always used high haloperidol doses (≥ 10 mg/d), which could have skewed the overall curve to the right. PET studies suggest that dopamine receptors are already fully blocked at 2-5 mg haloperidol per day.^35–37^ We tend to believe that the plateau dose of haloperidol in chronic patients with acute exacerbations of positive symptoms is already reached at 7.5 mg/d. We also observed some differences between formulations—oral, transdermal, and LAI—although these findings may be confounded by challenges in dose conversion between formulations.

Several additional limitations of this study should not be overlooked. First, although 165 studies were identified, only approximately 68% contributed data suitable for meta-analysis. Second, for some antipsychotics, no analyzable fixed-dose data were available. This limitation is especially important for clozapine, because it is considered to be the most efficacious antipsychotic drug, and for the muscarinic M_1_/M_4_ agonist xanomeline–trospium, because it is the first antipsychotic with a non-primarily dopaminergic mechanism of action.^5^ Third, for most drugs the data at higher dose ranges were limited, increasing uncertainty and potentially explaining some changes at the end of the curves. Fourth, substantial variance was observed for some agents (e.g., amisulpride, blonanserin, lumateperone, and quetiapine). Fifth, the predominant negative symptoms and pediatric subgroups included few studies, reducing the robustness and generalizability of findings. Sixth, many trials evaluated a single active drug arm versus placebo rather than using true dose-finding designs, which may have influenced the shape of the dose–response curves. Sensitivity analyses excluding these single-dose trials showed that estimate for haloperidol (see discussion above) and amisulpride in patients with predominant negative symptoms changed substantially, indicating instability in the pooled results. Seventh, different formulations (and in the pooled analyses different drugs), were combined using dose equivalence assumptions, which may introduce conversion-related inaccuracies. Eighth, dose–response relationships were assessed visually and should therefore be interpreted with caution. Finally, although only antipsychotic monotherapy trials were included, concomitant medication use in particular benzodiazepines and anticholinergics, but also beta-blockers, antidepressants or mood-stabilizers, was variably permitted. While randomization reduced systematic imbalance in terms of pre-trial medication which could be continued in the studies, residual confounding from newly administered adjunctive treatments and other unmeasured factors cannot be entirely excluded.

## Conclusion

The identified curves provide an empirical framework for understanding the range within which dose increases of antipsychotic drugs are associated with meaningful gains in symptom reduction. It is important to note that the present analysis focuses on efficacy outcomes and does not integrate adverse event data. Such information may assist clinicians in avoiding unnecessarily high doses once the efficacy plateau is reached. Avoiding unnecessarily high dose is particularly important because they can lead to excess side effects without additional benefits on symptom reductions; dose–response information on side-effects has been reported elsewhere.^10–13^ On the contrary, high doses, particularly of drug with strong dopamine-blockade can lead to secondary, drug-induced negatives symptoms due to blockade of the reward system or extrapyramidal side effects.

## Supplementary Material

supplementary_material_revised_ssbag077

## Data Availability

The full dataset is available from the study authors upon reasonable request.
