# Supplementary material for: Positive and Negative Symptoms Changes in Schizophrenia Patients on Antipsychotic Treatment: a Systematic Review and Dose–Response Meta-analysis
Source: Schizophr Bull. 2026 Aug 29;52(5):sbag077. doi: 10.1093/schbul/sbag077 (PMC13525547; doi:10.1093/schbul/sbag077)
Supplement: Supplementary file 1 [file supplementary_material_revised_ssbag077.docx]

**Supplementary information**

**Positive and Negative Symptoms Changes in Schizophrenia Patients on Antipsychotic Treatment: A Systematic Review and Dose-Response Meta-Analysis**

Xiao Lin, Spyridon Siafis, Jing Tian, Hui Wu, Christoph U. Correll, Schneider-Thoma Johannes, Leucht Stefan

Table of Contents

[Appendix 1: PRISMA checklist	3](#_Toc226462859)

[Appendix 2: PROSPERO registration and protocol	6](#_Toc226462860)

[Differences between protocol and review	6](#_Toc226462861)

[Appendix 3: Search strategy	7](#_Toc226462862)

[Appendix 4: PRISMA flow chart	8](#_Toc226462863)

[Appendix 5: Details about outcome parameters	10](#_Toc226462864)

[Appendix 6: Dose conversion	11](#_Toc226462865)

[Table S1: Dose conversion scheme	11](#_Toc226462866)

[Appendix 7: Description of included studies	13](#_Toc226462867)

[Table S2: Description of included studies	13](#_Toc226462868)

[Table S3: Summary characteristics of studies with usable data	53](#_Toc226462869)

[Appendix 8: Risk of bias assessment	54](#_Toc226462870)

[Assessments of the risk of bias of individual studies used in the meta-analysis	54](#_Toc226462871)

[Table S4: Risk of bias for individual studies assessed by Cochrane risk of bias tool 1^1^	54](#_Toc226462872)

[Summary of risk of bias of included studies used in the meta-analysis	59](#_Toc226462873)

[Figure S1: Summary of risk of bias of included studies assessed by Cochrane risk of bias tool 1	59](#_Toc226462874)

[Appendix 9: Summary of the statistical results of the meta-analysis	60](#_Toc226462875)

[Table S5: Statistical summary of the participants with acute exacerbation	60](#_Toc226462876)

[Table S6: Statistical summary of the participants with predominant negative symptoms	63](#_Toc226462877)

[Table S7: Statistical summary of the children or adolescent participants	64](#_Toc226462878)

[Appendix 10: Dose-Dependent Effects of Antipsychotics on Positive and Negative Symptoms in different patients groups (with CIs)	66](#_Toc226462879)

[Figure S2 Positive/negative symptoms change of individual antipsychotics in different dosages in adult participants with acute exacerbations of chronic schizophrenia	66](#_Toc226462880)

[Figure S3 Positive/negative symptoms change of individual antipsychotics in different dosages in participants with predominant negative symptoms of chronic schizophrenia	69](#_Toc226462881)

[Figure S4 Positive/negative symptoms change of individual antipsychotics in different dosages in child/ adolescent participants with acute exacerbations of chronic schizophrenia	70](#_Toc226462882)

[Appendix 11: Sensitivity analyses	72](#_Toc226462883)

[Figure S5 Dose-Dependent Effects of Antipsychotics on Positive and Negative Symptoms: Exclusion of Single-Dose vs. Placebo Comparisons	72](#_Toc226462884)

[Figure S6 Dose-Dependent Effects of Antipsychotics on Positive and Negative Symptoms: Exclusion of studies in treatment-resistant patients	74](#_Toc226462885)

[Figure S7 Dose-Dependent Effects of Antipsychotics on Positive and Negative Symptoms: Separate analyses of different formulations of antipsychotics.	75](#_Toc226462886)

[Figure S8 Olanzapine-equivalent dose–response curves across antipsychotics excluding haloperidol.	77](#_Toc226462887)

[Appendix 12: Heterogeneity assessments	78](#_Toc226462888)

[Heterogeneity assessments with the variance-partition-coefficient (VPC) for participants with acute exacerbation	78](#_Toc226462889)

[VPC for participants with predominant negative symptoms	80](#_Toc226462890)

[VPC for child/adolescent participants	80](#_Toc226462891)

[Appendix 13: Small-study effect and publication bias assessments	81](#_Toc226462892)

[Figure S9: Forest plot of Aripiprazole (all doses combined) compared to placebo	81](#_Toc226462893)

[Figure S10: Forest plot of Haloperidol (all doses combined) compared to placebo	81](#_Toc226462894)

[Figure S11: Forest plot of Olanzapine (all doses combined) compared to placebo	82](#_Toc226462895)

[Figure S12: Forest plot of Paliperidone (all doses combined) compared to placebo	83](#_Toc226462896)

[Figure S13: Forest plot of Risperidone (all doses combined) compared to placebo	83](#_Toc226462897)

[Figure S14: Funnel plot for Aripiprazole studies	85](#_Toc226462898)

[Figure S15: Funnel plot for Haloperidol studies	86](#_Toc226462899)

[Figure S16: Funnel plot for Olanzapine studies	87](#_Toc226462900)

[Figure S17: Funnel plot for Paliperidone studies	88](#_Toc226462901)

[Figure S18: Funnel plot for Risperidone studies	89](#_Toc226462902)

# Appendix 1: PRISMA checklist

| **Section and Topic** | **Item #** | **Checklist item** | **Location where item is reported** |
| --- | --- | --- | --- |
| **TITLE** | | |  |
| Title | 1 | Identify the report as a systematic review. | 1 |
| **ABSTRACT** | | |  |
| Abstract | 2 | See the PRISMA 2020 for Abstracts checklist. | 3-4 |
| **INTRODUCTION** | | |  |
| Rationale | 3 | Describe the rationale for the review in the context of existing knowledge. | 5-6 |
| Objectives | 4 | Provide an explicit statement of the objective(s) or question(s) the review addresses. | 5-6 |
| **METHODS** | | |  |
| Eligibility criteria | 5 | Specify the inclusion and exclusion criteria for the review and how studies were grouped for the syntheses. | 7-9 |
| Information sources | 6 | Specify all databases, registers, websites, organisations, reference lists and other sources searched or consulted to identify studies. Specify the date when each source was last searched or consulted. | 6-7, Appendix 3 |
| Search strategy | 7 | Present the full search strategies for all databases, registers and websites, including any filters and limits used. | 6-7, Appendix 3 |
| Selection process | 8 | Specify the methods used to decide whether a study met the inclusion criteria of the review, including how many reviewers screened each record and each report retrieved, whether they worked independently, and if applicable, details of automation tools used in the process. | 10-11 |
| Data collection process | 9 | Specify the methods used to collect data from reports, including how many reviewers collected data from each report, whether they worked independently, any processes for obtaining or confirming data from study investigators, and if applicable, details of automation tools used in the process. | 10-11 |
| Data items | 10a | List and define all outcomes for which data were sought. Specify whether all results that were compatible with each outcome domain in each study were sought (e.g. for all measures, time points, analyses), and if not, the methods used to decide which results to collect. | 10 |
|  | 10b | List and define all other variables for which data were sought (e.g. participant and intervention characteristics, funding sources). Describe any assumptions made about any missing or unclear information. | 7-8 |
| Study risk of bias assessment | 11 | Specify the methods used to assess risk of bias in the included studies, including details of the tool(s) used, how many reviewers assessed each study and whether they worked independently, and if applicable, details of automation tools used in the process. | 10-11 |
| Effect measures | 12 | Specify for each outcome the effect measure(s) (e.g. risk ratio, mean difference) used in the synthesis or presentation of results. | 9 |
| Synthesis methods | 13a | Describe the processes used to decide which studies were eligible for each synthesis (e.g. tabulating the study intervention characteristics and comparing against the planned groups for each synthesis (item #5)). | 7-9, Appendix 4 |
|  | 13b | Describe any methods required to prepare the data for presentation or synthesis, such as handling of missing summary statistics, or data conversions. | 10, Appendix 6 |
|  | 13c | Describe any methods used to tabulate or visually display results of individual studies and syntheses. | 10-11 |
|  | 13d | Describe any methods used to synthesize results and provide a rationale for the choice(s). If meta-analysis was performed, describe the model(s), method(s) to identify the presence and extent of statistical heterogeneity, and software package(s) used. | 10-11 |
|  | 13e | Describe any methods used to explore possible causes of heterogeneity among study results (e.g. subgroup analysis, meta-regression). | 10-11 |
|  | 13f | Describe any sensitivity analyses conducted to assess robustness of the synthesized results. | 10-11 |
| Reporting bias assessment | 14 | Describe any methods used to assess risk of bias due to missing results in a synthesis (arising from reporting biases). | 11 |
| Certainty assessment | 15 | Describe any methods used to assess certainty (or confidence) in the body of evidence for an outcome. | GRADE is not available for dose-response meta-analysis |
| **RESULTS** | | |  |
| Study selection | 16a | Describe the results of the search and selection process, from the number of records identified in the search to the number of studies included in the review, ideally using a flow diagram. | 11-12, Appendix 3-4 |
|  | 16b | Cite studies that might appear to meet the inclusion criteria, but which were excluded, and explain why they were excluded. | n.a. |
| Study characteristics | 17 | Cite each included study and present its characteristics. | Appendix 7 |
| Risk of bias in studies | 18 | Present assessments of risk of bias for each included study. | Appendix 8 |
| Results of individual studies | 19 | For all outcomes, present, for each study: (a) summary statistics for each group (where appropriate) and (b) an effect estimate and its precision (e.g. confidence/credible interval), ideally using structured tables or plots. | Appendix 9 |
| Results of syntheses | 20a | For each synthesis, briefly summarise the characteristics and risk of bias among contributing studies. | Appendix 8 |
|  | 20b | Present results of all statistical syntheses conducted. If meta-analysis was done, present for each the summary estimate and its precision (e.g. confidence/credible interval) and measures of statistical heterogeneity. If comparing groups, describe the direction of the effect. | 13-27, Appendix 10 |
|  | 20c | Present results of all investigations of possible causes of heterogeneity among study results. | 28 |
|  | 20d | Present results of all sensitivity analyses conducted to assess the robustness of the synthesized results. | 27, Appendix 11 |
| Reporting biases | 21 | Present assessments of risk of bias due to missing results (arising from reporting biases) for each synthesis assessed. | Appendix 8 |
| Certainty of evidence | 22 | Present assessments of certainty (or confidence) in the body of evidence for each outcome assessed. | GRADE is not available for dose-response meta-analysis |
| **DISCUSSION** | | |  |
| Discussion | 23a | Provide a general interpretation of the results in the context of other evidence. | 28 |
|  | 23b | Discuss any limitations of the evidence included in the review. | 34-35 |
|  | 23c | Discuss any limitations of the review processes used. | 34-35 |
|  | 23d | Discuss implications of the results for practice, policy, and future research. | 34-35 |
| **OTHER INFORMATION** | | |  |
| Registration and protocol | 24a | Provide registration information for the review, including register name and registration number, or state that the review was not registered. | 6 |
|  | 24b | Indicate where the review protocol can be accessed, or state that a protocol was not prepared. | 6 |
|  | 24c | Describe and explain any amendments to information provided at registration or in the protocol. | Appendix 2 |
| Support | 25 | Describe sources of financial or non-financial support for the review, and the role of the funders or sponsors in the review. | Role of the Funding source |
| Competing interests | 26 | Declare any competing interests of review authors. | Conflict of Interest |
| Availability of data, code and other materials | 27 | Report which of the following are publicly available and where they can be found: template data collection forms; data extracted from included studies; data used for all analyses; analytic code; any other materials used in the review. | n.a. Available upon reasonable request to the corresponding author |

*From:* Page MJ, McKenzie JE, Bossuyt PM, Boutron I, Hoffmann TC, Mulrow CD, et al. The PRISMA 2020 statement: an updated guideline for reporting systematic reviews. BMJ 2021;372:n71. doi: 10.1136/bmj.n71

# Appendix 2: PROSPERO registration and protocol

Registration number: CRD42020181467

Available from: <https://www.crd.york.ac.uk/prospero/display_record.php?ID=CRD42020181467>

## Differences between protocol and review

During the conduct of the project additional drugs (blonanserin, perospirone, olanzapine-samidorphan, and xanomeline–trospium) became licensed by the FDA. Therefore, we included them in the review, however, due to a lack of data, we could not analyze any of these drugs except blonanserin.

We pooled the different formulations of the same antipsychotic by transferring to oral dose in the primary analysis, but reported them separately in sensitivity analyses.

Sensitivity analyses were only applied in chronic schizophrenia patients with acute exacerbation, in patients with predominant symptoms, and in children/adolescents, since we had sparse data for first episode and no data for elderly patients.

In addition to clinical judgement and visual inspection, we also used the variance partition coefficient (VPC) to quantify heterogeneity.

# Appendix 3: Search strategy

1. Studies identified in previous work (153 studies)

2. An update search specifically for studies on blonanserin and perospirone (Inception of databases to March-06-2022) (125 reports) and olanzapine-samidorphan (Inception of databases to March-06-2022) (39 reports)

3. An update search in CSzG’s register (March-07- 2022 to July-26-2024) (359 reports)

(The antipsychotics such as blonanserin, perospirone, and olanzapine/samidorphan had not been included in our initial searches. Consequently, we conducted a targeted search for these drugs from inception of the register until March-06-2022 to ensure their inclusion.)

Studies identified in previous work

We have done exhausted searches for the previously published meta-analyses, from which we identified 153 eligible studies, the dose-response meta-analysis of antipsychotic drugs for acute schizophrenia[1], the network meta-analysis of acute treatment for schizophrenia [2], meta-analyses in patients with first episode [3], in treatment-resistant schizophrenia [4], predominant negative symptoms [5], the dose-response meta-analysis of antipsychotic-induced weight gain[6] and the extrapyramidal side-effects[7].

Details in the search strategies and study identification can be found in the respective reviews.

Search Results: 153 studies.

The search for Blonanserin, Perospirone and Olanzapine-Samidorphan

Date: March-06-2022

Strategy: *blonaserin*, *perospirone*,* olanzapine+samidorphan*

Study Records

Search Results: There were 164 reports.

The search in the Cochrane Schizophrenia Group’s Study-Based Register

Date: June-25-2023, January-24-2024, and July-26-2024

We searched the register of the Cochrane schizophrenia group for references added to the register between March-07-2022 to June-25-2023 reporting about studies indexed as investigating one of the following interventions:

*Amisulpride, Aripiprazole, Asenapine, Benperidol, Blonanserin, Brexpiprazole, Cariprazine, Chlorpromazine, Clopenthixol, Clozapine, Flupentixol, Fluphenazine, Fluspirilene, Haloperidol, Iloperidone, Levomepromazine, Loxapine, Lumateperone, Lurasidone, Molindone, Olanzapine, Olanzapine+Samidorphan, Paliperidone, Penfluridol, Perazine, Perospirone, Perphenazine, Pimozide, Quetiapine, Risperidone, Samidorphan, Sertindole, Sulpiride, Thioridazine, Tiotixene, Trifluoperazine, Ziprasidone, Zotepine, Zuclopenthixol*

January-24-2024 and July-26-2024 the drug “Xanomeline” was additionally added.

Search Results: There were 359 reports.

# Appendix 4: PRISMA flow chart

*:Broad selection criteria of our group’s database: We exclude *No randomized controlled trial (RCT) studies, duration less than 3 weeks, population less than 80% schizophrenia, studies conducted and published in China only, no usable comparison (studies investigating combinations of antipsychotics, studies which compared an included antipsychotic drug to another intervention which is not among the list of included antipsychotics or placebo, studies which compare an included antipsychotic to grouped drug groups such as “any FGA”(* *first-generation antipsychotics)).*

***: 15 studies found in the update search were already included in the previous dose-response meta-analyses of our group. In the update searches additional references to these studies were found.*

**References**

1.	Leucht, S., et al., *Dose-Response Meta-Analysis of Antipsychotic Drugs for Acute Schizophrenia.* American Journal of Psychiatry, 2020. **177**(4): p. 342-353.

2.	Huhn, M., et al., *Comparative efficacy and tolerability of 32 oral antipsychotics for the acute treatment of adults with multi-episode schizophrenia: a systematic review and network meta-analysis.* Lancet, 2019. **394**(10202): p. 939-951.

3.	Zhu, Y., et al., *How well do patients with a first episode of schizophrenia respond to antipsychotics: A systematic review and meta-analysis.* Eur Neuropsychopharmacol, 2017. **27**(9): p. 835-844.

4.	Samara, M.T., et al., *Efficacy, Acceptability, and Tolerability of Antipsychotics in Treatment-Resistant Schizophrenia A Network Meta-analysis.* Jama Psychiatry, 2016. **73**(3): p. 199-210.

5.	Krause, M., et al., *Antipsychotic drugs for patients with schizophrenia and predominant or prominent negative symptoms: a systematic review and meta-analysis.* European Archives of Psychiatry and Clinical Neuroscience, 2018. **268**(7): p. 625-639.

6.	Wu, H., et al., *Antipsychotic-induced akathisia in adults with acute schizophrenia: A systematic review and dose-response meta-analysis.* Eur Neuropsychopharmacol, 2023. **72**: p. 40-49.

7.	Siafis, S., et al., *Antipsychotic dose, dopamine D2 receptor occupancy and extrapyramidal side-effects: a systematic review and dose-response meta-analysis.* Mol Psychiatry, 2023. **28**(8): p. 3267-3277.

# Appendix 5: Details about outcome parameters

For positive symptoms, we considered all relevant scales and prioritized them in the following order: PANSS positive change, SAPS positive change, PANSS positive endpoint, SAPS positive endpoint, BPRS positive change/endpoint, BPRS core items, PANSS Marder positive factor score, and other published rating scales for schizophrenia's positive symptoms. Similarly, for negative symptoms, the prioritization included: PANSS negative change, SANS change, PANSS negative endpoint, SANS endpoint, BPRS negative change/endpoint, and other published rating scales for schizophrenia's negative symptoms.

# Appendix 6: Dose conversion

Table S1: Dose conversion scheme

| Drug formulation | Dose | Oral equivalence |
| --- | --- | --- |
| Aripiprazole lauroxil [1] | 441 mg/4 weeks | 10.71429 mg/d^b^ |
|  | 882 mg/4 weeks | 21.42857 mg/d^b^ |
| Aripiprazole monohydrate | 396.4 mg/4 weeks | 14.15714 mg/d^a^ |
| Asenapine HP3070 [2] | 3.8 mg/d | 10mg/d^b^ |
|  | 7.6 mg/d | 20mg/d^b^ |
| Blonanserin transdermal [3] | 40mg/d | 8mg/d^b^ |
|  | 80mg/d | 16mg/d^b^ |
| Olanzapine LAI (pamoate) | 210 mg/2 weeks | 15 mg/d^a^ |
|  | 300 mg/2 weeks | 21.42857 mg/d^a^ |
|  | 405 mg/4 weeks | 14.46429 mg/d^a^ |
| Paliperidone LAI [4] | 25 mg/4 weeks | 2 mg/d^b^ |
|  | 50 mg/4 weeks | 4 mg/d^b^ |
|  | 100 mg/4 weeks | 8 mg/d^b^ |
|  | 150 mg/4 weeks | 12 mg/d^b^ |
| Risperidone microspheres | 25 mg/2 weeks | 1.785714 mg/d^a^ |
|  | 50 mg/2 weeks | 3.571429 mg/d^a^ |
|  | 100 mg/2 weeks | 7.142857 mg/d^a^ |
| Risperidone RBP-7000 | 90 mg/4 weeks | 3.214286 mg/d^a^ |
|  | 120 mg/4 weeks | 4.285714 mg/d^a^ |
| Risperidone ISM | 75mg/4 weeks | 3mg/d^c^ |
|  | 100mg/4 weeks | 4mg/d^c^ |

Long injectable doses converted to daily oral equivalences in these ways: ^a^ dividing the LAI doses by the number of days; ^b^ according to the published dose equivalences; ^c^ dose equivalence recommendations from Laboratorios Farmacéuticos ROVI (personal contact).

**References**

1.	Rothe, P.H., S. Heres, and S. Leucht, *Dose equivalents for second generation long-acting injectable antipsychotics: The minimum effective dose method.* Schizophrenia Research, 2018. **193**: p. 23-28.

2.	Secuado (asenapine) [prescribing information]. Miami, FL: Noven Therapeutics, LLC; 2019.

3.	Tomita, Y., et al., *Prediction of Corresponding Dose of Transdermal Blonanserin to Oral Dose Based on Dopamine D2 Receptor Occupancy: Unique Characteristics of Blonanserin Transdermal Patch.* J Clin Psychopharmacol, 2022. **42**(3): p. 260-269.

4.	Gopal, S., et al., *Practical guidance for dosing and switching paliperidone palmitate treatment in patients with schizophrenia.* Current Medical Research and Opinion, 2009. **26**(2): p. 377-387.

# Appendix 7: Description of included studies

### Table S2: Description of included studies

| **Study name** | **Duration (weeks)** | **Diagnosis** | **Route** | **Setting** | **Intervention** | **Dosing interval** | **Dose (mg)** | **N** | **N male** | **Mean age (years)** | **Duration of illness (years)** |
| --- | --- | --- | --- | --- | --- | --- | --- | --- | --- | --- | --- |
| Arvanitis 1997 [1] | 6 | acute exacerbation of chronic or subchronic schizophrenia (DSM-III-R) | oral | inpatient | Haloperidol | daily | 12 | 52 | 42 | 37 | 16 |
|  |  |  |  |  | Placebo | daily | 0 | 51 | 41 | 36 | 14 |
|  |  |  |  |  | Quetiapine | daily | 75 | 53 | 39 | 37 | 15 |
|  |  |  |  |  | Quetiapine | daily | 150 | 48 | 39 | 38 | 16 |
|  |  |  |  |  | Quetiapine | daily | 300 | 52 | 37 | 38 | 16 |
|  |  |  |  |  | Quetiapine | daily | 600 | 51 | 38 | 39 | 15 |
|  |  |  |  |  | Quetiapine | daily | 750 | 54 | 38 | 35 | 13 |
| Beasley 1996a [2] | 6 | schizophrenia (DSM-III-R) | oral | inpatient | Placebo | daily | 0 | 50 | 33 | 36 | 14 |
|  |  |  |  |  | Olanzapine | daily | 1 | 52 | 40 | 38 | 16 |
|  |  |  |  |  | Olanzapine | daily | 10 | 50 | 37 | 39 | 18 |
| Beasley 1996b [3] | 6 | schizophrenia with an acute exacerbation (DSM-III-R) | oral | inpatient | Haloperidol | daily | 16.40 | 69 | 62 | 36 | 15 |
|  |  |  |  |  | Placebo | daily | 0 | 68 | 62 | 35 | 13 |
|  |  |  |  |  | Olanzapine | daily | 6.60 | 65 | 60 | 35.7 | 13.87 |
|  |  |  |  |  | Olanzapine | daily | 11.60 | 64 | 56 | 37.29 | 15.01 |
|  |  |  |  |  | Olanzapine | daily | 16.30 | 69 | 54 | 35.93 | 13.25 |
| Beasley 1997 [4] | 6 | schizophrenia (DSM-III-R) | oral | inpatient | Haloperidol | daily | 17.60 | 81 | 48 | 36 | 12.6 |
|  |  |  |  |  | Olanzapine | daily | 1 | 88 | 58 | 34 | 10.1 |
|  |  |  |  |  | Olanzapine | daily | 6.70 | 87 | 57 | 34 | 9.4 |
|  |  |  |  |  | Olanzapine | daily | 11.30 | 86 | 55 | 36 | 11.4 |
|  |  |  |  |  | Olanzapine | daily | 16.40 | 89 | 57 | 37 | 12.7 |
| Berger 2008 [5] | 4 | schizophreniform psychosis, schizophrenia, schizoaffective disorder, delusional disorder, major depression with psychotic features, or psychosis not otherwise specified (DSM-IV) | oral | both | Quetiapine | daily | 200 | 69 | 44 | 19.7 | 0.63 |
|  |  |  |  |  | Quetiapine | daily | 400 | 72 | 41 | 19 | 0.62 |
| Boyer 1995 [6] | 6 | schizophrenia predominant negative symptoms (DSM-III and Andreasen's criteria for negative schizophrenia) | oral | inpatient | Placebo | daily | 0 | 34 | 23 | 30.7 | 9.75 |
|  |  |  |  |  | Amisulpride | daily | 100 | 34 | 20 | 32.5 | 9.83 |
|  |  |  |  |  | Amisulpride | daily | 300 | 36 | 22 | 34.1 | 12.08 |
| Bugarski-Kirola 2014 [7] | 4 | acute exacerbation of schizophrenia (DSM-IV) | oral | inpatient | Placebo | daily | 0 | 79 | 58 | 37.8 | 12.5 |
|  |  |  |  |  | Olanzapine | daily | 15 | 63 | 46 | 40.3 | 13.9 |
| Cantillon 2014 [8] | 4 | subjects with an acute exacerbation of schizophrenia or schizoaffective disorder (DSM-IV-TR) | oral | n.i. | Placebo | daily | 0 | 38 | 27 | 36 | n.i. |
|  |  |  |  |  | Aripiprazole | daily | 15 | 20 | 18 | 35 | n.i. |
| Canuso 2010b [9] | 6 | acute exacerbation of schizoaffective disorder (DSM-IV) | oral | inpatient | Placebo | daily | 0 | 107 | 67 | 37.1 | 10.5 |
|  |  |  |  |  | Paliperidone | daily | 5.70 | 109 | 70 | 38.1 | 11.7 |
|  |  |  |  |  | Paliperidone | daily | 11.60 | 100 | 64 | 36.5 | 11.2 |
| Casey 2008 [10] | 6 | patients with an acute exacerbation of schizophrenia (DSM-IV-TR) | oral | inpatient | Placebo | daily | 0 | 119 | 88 | 40.8 | n.i. |
|  |  |  |  |  | Risperidone | daily | 6 | 120 | 94 | 41.1 | n.i. |
| Chouinard 1993 [11] | 8 | schizophrenia (DSM-III-R) | oral | inpatient | Haloperidol | daily | 20 | 21 | n.i. | n.i. | n.i. |
|  |  |  |  |  | Placebo | daily | 0 | 22 | n.i. | n.i. | n.i. |
|  |  |  |  |  | Risperidone | daily | 2 | 24 | n.i. | n.i. | n.i. |
|  |  |  |  |  | Risperidone | daily | 6 | 22 | n.i. | n.i. | n.i. |
|  |  |  |  |  | Risperidone | daily | 10 | 22 | n.i. | n.i. | n.i. |
|  |  |  |  |  | Risperidone | daily | 16 | 24 | n.i. | n.i. | n.i. |
| Citrome 2021 [12] | 6 | schizophrenia (DSM-5) | transdermal | inpatient | Placebo | daily | 0 | 206 | 131 | 42.1 | 15.4 |
|  |  |  |  |  | Asenapine | daily | 3.80 | 205 | 131 | 41.5 | 15.5 |
|  |  |  |  |  | Asenapine | daily | 7.60 | 206 | 111 | 42.3 | 16.1 |
| Cooper 2000a^b^[13] | 8 | schizophrenia (DSM-III-R) | oral | both | Placebo | daily | 0 | 53 | 37 | 36.3 | 10.28 |
|  |  |  |  |  | Zotepine | daily | 240.57 | 53 | 37 | 39.6 | 10.78 |
| Correll 2015 [14] | 6 | schizophrenia (DSM-IV-TR) | oral | inpatient | Placebo | daily | 0 | 184 | 118 | 39.7 | 12.3 |
|  |  |  |  |  | Brexpiprazole | daily | 0.25 | 90 | 61 | 40.5 | 13 |
|  |  |  |  |  | Brexpiprazole | daily | 2 | 182 | 111 | 39.6 | 13 |
|  |  |  |  |  | Brexpiprazole | daily | 4 | 180 | 111 | 40.8 | 12.8 |
| Correll 2020 [15] | 4 | schizophrenia (DSM-5) | oral | inpatient | Placebo | daily | 0 | 150 | 123 | 41.4 | 17.4 |
|  |  |  |  |  | Lumateperone | daily | 40 | 150 | 113 | 43.5 | 17 |
|  |  |  |  |  | Lumateperone | daily | 60 | 150 | 110 | 42.4 | 16.5 |
| Correll 2020b [16] | 12 | schizophrenia (DSM-5) | depot | both | Placebo | every 4 weeks | 0 | 147 | 98 | 40.5 | 14.6 |
|  |  |  |  |  | Risperidone | every 4 weeks | 75 | 145 | 98 | 42.5 | 16.1 |
|  |  |  |  |  | Risperidone | every 4 weeks | 100 | 146 | 97 | 42.9 | 15.9 |
| Corrigan 2004 [17] | 6 | schizophrenia (DSM-IV) | oral | inpatient | Placebo | daily | 0 | 87 | 63 | 37.2 | 14.2 |
|  |  |  |  |  | Olanzapine | daily | 15 | 93 | 59 | 36.8 | 12.3 |
| Cutler 2006 [18] | 6 | schizophrenia acute relapse (DSM-IV) | oral | inpatient | Placebo | daily | 0 | 88 | 72 | 42.9 | 18.3 |
|  |  |  |  |  | Aripiprazole | daily | 2 | 93 | 74 | 40.7 | 15.3 |
|  |  |  |  |  | Aripiprazole | daily | 5 | 92 | 70 | 40.9 | 16.9 |
|  |  |  |  |  | Aripiprazole | daily | 10 | 94 | 72 | 40 | 14.8 |
| Cutler 2008 [19] | 4 | Schizophrenia (DSM-IV) | oral | inpatient | Iloperidone | daily | 24 | 303 | 245 | 39.5 | n.i. |
|  |  |  |  |  | Placebo | daily | 0 | 152 | 114 | 40.7 | n.i. |
|  |  |  |  |  | Ziprasidone | daily | 160 | 151 | 113 | 40 | n.i. |
| Cutler 2008a [20] | 6 | Schizophrenia (DSM-IV) | oral | both | Placebo | daily | 0 | 117 | 77 | 42.5 | 18.3 |
|  |  |  |  |  | Quetiapine | daily | 400 | 114 | 79 | 42.1 | 18.8 |
|  |  |  |  |  | Quetiapine | daily | 600 | 105 | 82 | 41.2 | 18 |
|  |  |  |  |  | Quetiapine | daily | 800 | 113 | 82 | 40.2 | 16.3 |
|  |  |  |  |  | Quetiapine | daily | 800 | 116 | 69 | 40.8 | 16.9 |
| Daniel 1999 [21] | 6 | acute exacerbation of (sub-) chronic schizophrenia or schizoaffective disorder (DSM-III-R) | oral | inpatient | Placebo | daily | 0 | 92 | 63 | 37.2 | 14.7 |
|  |  |  |  |  | Ziprasidone | daily | 80 | 106 | 75 | 36.8 | 13.6 |
|  |  |  |  |  | Ziprasidone | daily | 160 | 104 | 77 | 35.8 | 14.4 |
| Danion 1999 [22] | 12 | schizophrenia of residual type (DSM-III-R) | oral | both | Placebo | daily | 0 | 83 | 52 | 35 | 9.3 |
|  |  |  |  |  | Amisulpride | daily | 50 | 84 | 52 | 33.4 | 9.4 |
|  |  |  |  |  | Amisulpride | daily | 100 | 75 | 50 | 35.7 | 10.1 |
| Davidson 2007 [23] | 6 | schizophrenia acute episode (DSM-IV) | oral | inpatient | Olanzapine | daily | 10 | 128 | 96 | 36.5 | 11.9 |
|  |  |  |  |  | Placebo | daily | 0 | 123 | 83 | 37.3 | 12.8 |
|  |  |  |  |  | Paliperidone | daily | 3 | 127 | 77 | 36.3 | 10.6 |
|  |  |  |  |  | Paliperidone | daily | 9 | 125 | 79 | 36.2 | 11 |
|  |  |  |  |  | Paliperidone | daily | 15 | 115 | 73 | 37.6 | 12.4 |
| Durgam 2014 [24] | 6 | acute exacerbation of schizophrenia (DSM-IV-TR) | oral | inpatient | Cariprazine | daily | 1.50 | 145 | 93 | 36.8 | 11.4 |
|  |  |  |  |  | Cariprazine | daily | 3 | 146 | 107 | 37.1 | 11.2 |
|  |  |  |  |  | Cariprazine | daily | 4.50 | 147 | 103 | 35.8 | 11.1 |
|  |  |  |  |  | Placebo | daily | 0 | 151 | 101 | 36 | 11.6 |
|  |  |  |  |  | Risperidone | daily | 4 | 140 | 98 | 36.5 | 12.3 |
| Durgam 2015 [25] | 6 | Schizophrenia (DSM-IV-TR) | oral | inpatient | Aripiprazole | daily | 10 | 152 | 94 | 39.3 | 12.4 |
|  |  |  |  |  | Cariprazine | daily | 3 | 155 | 99 | 37.9 | 12.4 |
|  |  |  |  |  | Cariprazine | daily | 6 | 157 | 100 | 38.6 | 11.7 |
|  |  |  |  |  | Placebo | daily | 0 | 153 | 97 | 38.2 | 12.5 |
| Durgam 2016a [26] | 6 | schizophrenia (DSM-IV-TR) | oral | inpatient | Placebo | daily | 0 | 130 | 103 | 41.1 | 17.7 |
|  |  |  |  |  | Cariprazine | daily | 3.83 | 128 | 105 | 40.3 | 17.2 |
|  |  |  |  |  | Cariprazine | daily | 8.70 | 134 | 101 | 42.4 | n.i. |
| Egan 2013 [27] | 4 | schizophrenia, acutely psychotic (DSM-IV-TR) | oral | inpatient | Placebo | daily | 0 | 83 | 53 | 36.4 | 11.2 |
|  |  |  |  |  | Olanzapine | daily | 15 | 47 | 24 | 36.1 | 10.7 |
| Fabre 1995 [28] | 3 | (sub-)chronic schizophrenia (DSM-III-R) | oral | inpatient | Placebo | daily | 0 | 4 | 4 | 35 | 12 |
|  |  |  |  |  | Quetiapine | daily | 137.50 | 8 | 8 | 34 | 12 |
| Findling 2008 [29] | 6 | Schizophrenia (DSM-IV) | oral | both | Placebo | daily | 0 | 100 | 61 | 15.4 | 1.5 |
|  |  |  |  |  | Aripiprazole | daily | 9.80 | 100 | 45 | 15.6 | 1.4 |
|  |  |  |  |  | Aripiprazole | daily | 28.90 | 102 | 65 | 15.4 | 1.3 |
| Findling 2012 [30] | 6 | Schizophrenia (DSM-IV-TR) | oral | both | Placebo | daily | 0 | 75 | 42 | 15.34 | 2.22 |
|  |  |  |  |  | Quetiapine | daily | 400 | 73 | 43 | 15.45 | 2.34 |
|  |  |  |  |  | Quetiapine | daily | 800 | 74 | 44 | 15.45 | 2.54 |
| Findling 2015 [31] | 8 | schizophrenia (paranoid, disorganized, or undifferentiated subtype) (DSM-IV-TR) | oral | n.i. | Placebo | daily | 0 | 102 | 62 | 15.4 | n.i. |
|  |  |  |  |  | Asenapine | daily | 5 | 98 | 62 | 15.2 | 2 |
|  |  |  |  |  | Asenapine | daily | 10 | 106 | 67 | 15.4 | n.i. |
| Garcia 2009 [32] | 6 | acute exacerbation of schizophrenia (DSM-IV-TR) | oral | inpatient | Placebo | daily | 0 | 64 | 40 | 38.6 | n.i. |
|  |  |  |  |  | Blonanserin | daily | 2.50 | 61 | 33 | 38.3 | n.i. |
|  |  |  |  |  | Blonanserin | daily | 5 | 58 | 40 | 37 | n.i. |
|  |  |  |  |  | Blonanserin | daily | 10 | 64 | 35 | 38.4 | n.i. |
|  |  |  |  |  | Haloperidol | daily | 10 | 60 | 35 | 38.1 | n.i. |
| Goff 1998^a^[33] | 4 | chronic or subchronic schizophrenia or schizoaffective disorder (DSM-III-R) | oral | inpatient | Haloperidol | daily | 15 | 17 | 16 | 35.5 | 13.6 |
|  |  |  |  |  | Ziprasidone | daily | 4 | 19 | 17 | 41.7 | 18.3 |
|  |  |  |  |  | Ziprasidone | daily | 10 | 17 | 16 | 39.2 | n.i. |
|  |  |  |  |  | Ziprasidone | daily | 40 | 17 | 16 | 38.1 | n.i. |
|  |  |  |  |  | Ziprasidone | daily | 160 | 20 | 19 | 41.7 | 18.3 |
| Goff 2013 [34] | 8 | schizophrenia or schizo-affective disorder (DSM-IV) | oral | both | Ziprasidone | daily | 160 | 37 | 25 | 41 | 16.5 |
|  |  |  |  |  | Ziprasidone | daily | 320 | 38 | 27 | 39.2 | 13.7 |
| Goldman 2017 [35] | 6 | schizophrenia (DSM-IV-TR) | oral | inpatient | Placebo | daily | 0 | 113 | 41 | 15.3 | 2.2 |
|  |  |  |  |  | Lurasidone | daily | 40 | 108 | 43 | 15.5 | 2.1 |
|  |  |  |  |  | Lurasidone | daily | 80 | 106 | 34 | 15.3 | 2.4 |
| Gopal 2010 [36] | 13 | schizophrenia (DSM-IV) | depot | inpatient | Placebo | every 4 weeks | 0 | 136 | 94 | 41 | 15 |
|  |  |  |  |  | Paliperidone | every 4 weeks | 50 | 94 | 65 | 39 | 14 |
|  |  |  |  |  | Paliperidone | every 4 weeks | 100 | 97 | 61 | 39 | 14 |
|  |  |  |  |  | Paliperidone | every 4 weeks | 150 | 30 | 22 | 41 | 17 |
| Hale 2000 [37] | 8 | schizophrenia (DSM-III-R) | oral | inpatient | Haloperidol | daily | 10 | 125 | 84 | 36.5 | 9.5 |
|  |  |  |  |  | Sertindole | daily | 8 | 120 | 83 | 34.2 | 7.2 |
|  |  |  |  |  | Sertindole | daily | 16 | 127 | 84 | 34.3 | 6.3 |
|  |  |  |  |  | Sertindole | daily | 20 | 128 | 72 | 35.2 | 9.2 |
|  |  |  |  |  | Sertindole | daily | 24 | 117 | 77 | 35 | 8 |
| Hass 2009 [38] | 8 | schizophrenia (DSM-IV) | oral | inpatient | Risperidone | daily | 0.40 | 132 | 80 | 15.6 | 0.3 |
|  |  |  |  |  | Risperidone | daily | 4 | 125 | 65 | 15.6 | 0.3 |
| Higuchi 2019a [39] | 6 | schizophrenia with disorganized, paranoid, or undifferentiated subtypes (DSM-IV-TR) | oral | inpatient | Placebo | daily | 0 | 152 | 89 | 42.6 | 15.36 |
|  |  |  |  |  | Lurasidone | daily | 40 | 150 | 82 | 42.1 | 13.95 |
|  |  |  |  |  | Lurasidone | daily | 80 | 155 | 81 | 43.6 | 15.68 |
| Higuchi 2019b [40] | 6 | schizophrenia (DSM-IV) | oral | inpatient | Lurasidone | daily | 40 | 131 | 75 | 45.6 | n.i. |
|  |  |  |  |  | Lurasidone | daily | 80 | 131 | 82 | 45.7 | n.i. |
|  |  |  |  |  | Placebo | daily | 0 | 133 | 72 | 46 | n.i. |
|  |  |  |  |  | Risperidone | daily | 4 | 65 | 35 | 44.8 | n.i. |
| Hirayasu 2010 [41] | 6 | schizophrenia with acute symptoms (DSM-IV) | oral | both | Olanzapine | daily | 10 | 47 | 19 | 46.2 | 17.92 |
|  |  |  |  |  | Paliperidone | daily | 6 | 136 | 70 | 44 | 17.92 |
|  |  |  |  |  | Placebo | daily | 0 | 138 | 72 | 46.2 | 17.92 |
| Honer 2010 [42] | 8 | schizophrenia or schizoaffective disorder (DSM-IV) | oral | both | Quetiapine | daily | 799 | 43 | 32 | 37.9 | n.i. |
|  |  |  |  |  | Quetiapine | daily | 1144 | 88 | 58 | 40.6 | n.i. |
| Ishigooka 2018 [43] | 6 | schizophrenia (DSM-IV-TR) | oral | inpatient | Placebo | daily | 0 | 116 | 51 | 45 | 17.4 |
|  |  |  |  |  | Brexpiprazole | daily | 1 | 115 | 51 | 44.7 | 17.1 |
|  |  |  |  |  | Brexpiprazole | daily | 2 | 115 | 61 | 43.3 | 14.6 |
|  |  |  |  |  | Brexpiprazole | daily | 4 | 113 | 55 | 44.1 | 16.8 |
| Iwata 2019 [44] | 6 | schizophrenia (DSM-V) | transdermal | inpatient | Placebo | daily | 0 | 190 | 113 | 41.5 | 13.9 |
|  |  |  |  |  | Blonanserin | daily | 40 | 196 | 116 | 40.7 | 13.8 |
|  |  |  |  |  | Blonanserin | daily | 80 | 194 | 115 | 40.6 | 13.8 |
| Iyo 2021 [45] | 6 | schizophrenia (DSM-IV-TR) | oral | both | Placebo | daily | 0 | 236 | 121 | 39.3 | 10.4 |
|  |  |  |  |  | Lurasidone | daily | 40 | 247 | 120 | 41 | 10.6 |
| Kahn 2007 [46] | 6 | schizophrenia (DSM-IV) | oral | both | Placebo | daily | 0 | 118 | 67 | 34.1 | 8 |
|  |  |  |  |  | Quetiapine | daily | 400 | 123 | 69 | 34.4 | 8 |
|  |  |  |  |  | Quetiapine | daily | 400 | 113 | 78 | 34.1 | 8.8 |
|  |  |  |  |  | Quetiapine | daily | 600 | 113 | 61 | 34.2 | 8.9 |
|  |  |  |  |  | Quetiapine | daily | 800 | 121 | 70 | 34.4 | 8 |
| Kane 2002 [47] | 4 | schizophrenia or schizoaffective disorder, acute relapse (DSM-IV) | oral | inpatient | Placebo | daily | 0 | 106 | 74 | 38.5 | 16 |
|  |  |  |  |  | Aripiprazole | daily | 15 | 102 | 76 | 37.8 | 16 |
|  |  |  |  |  | Aripiprazole | daily | 30 | 102 | 70 | 39.3 | 17.2 |
|  |  |  |  |  | Haloperidol | daily | 10 | 104 | 68 | 38.9 | 16 |
| Kane 2003 [48] | 12 | schizophrenia (DSM-IV) | depot | both | Placebo | every 2 weeks | 0 | 98 | 80 | 37.7 | n.i. |
|  |  |  |  |  | Risperidone | every 2 weeks | 25 | 99 | 68 | 38.9 | n.i. |
|  |  |  |  |  | Risperidone | every 2 weeks | 50 | 103 | 84 | 36.2 | n.i. |
|  |  |  |  |  | Risperidone | every 2 weeks | 75 | 100 | 68 | 38.1 | n.i. |
| Kane 2007b [49] | 6 | acute episode of schizophrenia (DSM-IV) | oral | inpatient | Placebo | daily | 0 | 127 | 66 | 37.9 | 9.9 |
|  |  |  |  |  | Olanzapine | daily | 10 | 128 | 60 | 36.3 | 9.8 |
|  |  |  |  |  | Paliperidone | daily | 6 | 123 | 62 | 37 | 10.9 |
|  |  |  |  |  | Paliperidone | daily | 9 | 122 | 72 | 38.5 | 10.6 |
|  |  |  |  |  | Paliperidone | daily | 12 | 130 | 68 | 36 | 9.5 |
| Kane 2010a [50] | 6 | schizophrenia (DSM-IV-TR) | oral | inpatient | Placebo | daily | 0 | 123 | 64 | 40.1 | n.i. |
|  |  |  |  |  | Asenapine | daily | 10 | 114 | 75 | 38 | n.i. |
|  |  |  |  |  | Asenapine | daily | 20 | 106 | 67 | 37.1 | n.i. |
|  |  |  |  |  | Haloperidol | daily | 8 | 115 | 63 | 39 | n.i. |
| Kane 2014 [51] | 12 | schizophrenia (DSM-IV-TR) | depot | inpatient | Placebo | every 4 weeks | 0 | 172 | 139 | 42.7 | 18.7 |
|  |  |  |  |  | Aripiprazole | every 4 weeks | 396.4 | 168 | 130 | 42.1 | 17.7 |
| Kane 2015a [52] | 6 | schizophrenia (DSM-IV-TR) | oral | inpatient | Placebo | daily | 0 | 184 | 111 | 39.3 | 13.7 |
|  |  |  |  |  | Brexpiprazole | daily | 1 | 120 | 77 | 39.1 | 12.8 |
|  |  |  |  |  | Brexpiprazole | daily | 2 | 186 | 122 | 36.9 | 11.7 |
|  |  |  |  |  | Brexpiprazole | daily | 4 | 184 | 113 | 38.6 | 13.1 |
| Kane 2015b [53] | 6 | schizophrenia (DSM-IV-TR) | oral | inpatient | Placebo | daily | 0 | 147 | 110 | 36.7 | 11 |
|  |  |  |  |  | Cariprazine | daily | 5.58 | 151 | 118 | 36.6 | 11.3 |
|  |  |  |  |  | Cariprazine | daily | 8.39 | 148 | 113 | 35.5 | 9.9 |
| Keck 1998 [54] | 4 | acute exacerbation of subchronic or chronic schizophrenia or schizoaffective disorder (DSM-III-R) | oral | inpatient | Placebo | daily | 0 | 48 | 41 | 39 | 17.3 |
|  |  |  |  |  | Ziprasidone | daily | 40 | 44 | 30 | 40.5 | 17.2 |
|  |  |  |  |  | Ziprasidone | daily | 120 | 47 | 39 | 38.8 | 14.6 |
| King 1998^b^ [55] | 6 | hospitalised men and women with acute exacerbation of chronic or subchronic schizophrenia (DSM-III-R) | oral | inpatient | Quetiapine | daily | 50 | 209 | 135 | 37 | 11 |
|  |  |  |  |  | Quetiapine | daily | 450 | 200 | 128 | 34 | 10 |
|  |  |  |  |  | Quetiapine | daily | 450 | 209 | 146 | 36 | 10 |
| Kinon 2006c [56] | 8 | schizophrenia or schizoaffective disorder (DSM-IV) | oral | both | Olanzapine | daily | 10 | 199 | 134 | 41.16 | 17.96 |
|  |  |  |  |  | Olanzapine | daily | 20 | 200 | 135 | 40.85 | 17.13 |
|  |  |  |  |  | Olanzapine | daily | 40 | 200 | 139 | 41.6 | 17.63 |
| Kinon 2011 [57] | 4 | schizophrenia (DSM-IV) | oral | inpatient | Placebo | daily | 0 | 122 | 70 | 38.9 | 12.5 |
|  |  |  |  |  | Olanzapine | daily | 15 | 62 | 34 | 41.7 | 15 |
| Kinoshita 2016 [58] | 6 | schizophrenia of paranoid, disorganized, catatonic, or undifferentiated (295.90) subtype (DSM-IV-TR) | oral | inpatient | Placebo | daily | 0 | 174 | 81 | 41.11 | n.i. |
|  |  |  |  |  | Asenapine | daily | 10 | 176 | 75 | 41.41 | n.i. |
|  |  |  |  |  | Asenapine | daily | 20 | 182 | 99 | 41.72 | n.i. |
| Kramer 2010 [59] | 9 | schizophrenia (DSM-IV) | depot | inpatient | Placebo | every 4 weeks | 0 | 84 | 39 | 40 | 12 |
|  |  |  |  |  | Paliperidone | every 4 weeks | 50 | 79 | 41 | 40 | 13 |
|  |  |  |  |  | Paliperidone | every 4 weeks | 100 | 84 | 42 | 37 | 12 |
| Landbloom 2016 [60] | 6 | schizophrenia of paranoid, disorganized, or undifferentiated subtype (DSM-IV-TR) | oral | both | Placebo | daily | 0 | 103 | 54 | 41.4 | n.i. |
|  |  |  |  |  | Asenapine | daily | 5 | 98 | 58 | 41.5 | n.i. |
|  |  |  |  |  | Asenapine | daily | 10 | 113 | 69 | 39.1 | n.i. |
|  |  |  |  |  | Olanzapine | daily | 15 | 46 | 28 | 40.8 | n.i. |
| Lauriello 2008 [61] | 8 | schizophrenia (DSM-IV or DSM-IV-TR) | depot | inpatient | Placebo | every 4 weeks | 0 | 98 | 61 | 42.6 | 18.8 |
|  |  |  |  |  | Olanzapine | every 2 weeks | 210 | 106 | 79 | 39.8 | 16.3 |
|  |  |  |  |  | Olanzapine | every 2 weeks | 300 | 100 | 72 | 41.5 | 18 |
|  |  |  |  |  | Olanzapine | every 4 weeks | 405 | 100 | 73 | 39.5 | 16.7 |
| Lecrubier 2006 [62] | 26 | schizophrenia, residual, disorganised or catatonic (DSM-IV) | oral | both | Placebo | daily | 0 | 34 | 22 | 38.2 | 15.42 |
|  |  |  |  |  | Amisulpride | daily | 150 | 70 | 50 | 37.8 | 12.33 |
|  |  |  |  |  | Olanzapine | daily | 5 | 70 | 42 | 38.1 | 10.08 |
|  |  |  |  |  | Olanzapine | daily | 20 | 70 | 52 | 36.4 | 11.08 |
| Liebermann 2015 [63] | 4 | Schizophrenia (DSM-IV-TR) | oral | inpatient | Placebo | daily | 0 | 85 | 65 | 40.5 | 16.7 |
|  |  |  |  |  | Lumateperone | daily | 60 | 84 | 66 | 38.3 | 16 |
|  |  |  |  |  | Lumateperone | daily | 120 | 84 | 72 | 41.1 | 17 |
|  |  |  |  |  | Risperidone | daily | 4 | 82 | 73 | 40.7 | 15.2 |
| Lindenmayer 2008 [64] | 6 | schizophrenia (DSM-IV) | oral | inpatient | Placebo | daily | 0 | 84 | 65 | 38.2 | 14.7 |
|  |  |  |  |  | Quetiapine | daily | 300 | 90 | 68 | 39.8 | 16.8 |
|  |  |  |  |  | Quetiapine | daily | 600 | 86 | 63 | 40.6 | 14.7 |
|  |  |  |  |  | Quetiapine | daily | 300 | 91 | 67 | 39.1 | 16.2 |
|  |  |  |  |  | Quetiapine | daily | 600 | 92 | 66 | 38.9 | 16.1 |
|  |  |  |  |  | Quetiapine | daily | 800 | 89 | 73 | 37.8 | 15.1 |
| Lindenmayer 2011 [65] | 8 | Schizophrenia or schizoaffective disorder (DSM-IV-TR) | oral | inpatient | Quetiapine | daily | 600 | 31 | 27 | 40.97 | 21.55 |
|  |  |  |  |  | Quetiapine | daily | 1200 | 29 | 28 | 39.28 | 18.11 |
| Litman 2016 [66] | 4 | schizophrenia (DSM-IV) | oral | both | Placebo | daily | 0 | 55 | 44 | 36.9 | n.i. |
|  |  |  |  |  | Risperidone | daily | 4 | 31 | 26 | 39 | n.i. |
| Litmann 2014 [67] | 4 | schizophrenia, symptomatic patients but medically stable (DSM-IV) | oral | both | Placebo | daily | 0 | 41 | 39 | 40.2 | n.i. |
|  |  |  |  |  | Olanzapine | daily | 15 | 22 | 22 | 35.3 | n.i. |
| Loebel 2013 [68] | 6 | acute exacerbation of schizophrenia (DSM-IV-TR) | oral | inpatient | Placebo | daily | 0 | 120 | 77 | 37.4 | 11.3 |
|  |  |  |  |  | Lurasidone | daily | 80 | 125 | 96 | 36.2 | 11.1 |
|  |  |  |  |  | Lurasidone | daily | 160 | 121 | 82 | 37.9 | 11.8 |
|  |  |  |  |  | Quetiapine | daily | 600 | 120 | 77 | 37.4 | 12.4 |
| Loo 1997 [69] | 26 | schizophrenia with predominant negative symptoms (DSM-III-R) | oral | n.i. | Placebo | daily | 0 | 72 | 54 | 36 | 10.7 |
|  |  |  |  |  | Amisulpride | daily | 100 | 69 | 46 | 33 | 9.7 |
| Marder 1994 [70] | 8 | schizophrenia (DSM-III-R) | oral | inpatient | Haloperidol | daily | 20 | 66 | 60 | 38 | 15.4 |
|  |  |  |  |  | Placebo | daily | 0 | 66 | 57 | 37.1 | 15.2 |
|  |  |  |  |  | Risperidone | daily | 2 | 63 | 54 | 39.3 | 17.1 |
|  |  |  |  |  | Risperidone | daily | 6 | 64 | 55 | 37.5 | 15.8 |
|  |  |  |  |  | Risperidone | daily | 10 | 65 | 61 | 36.2 | 15.6 |
|  |  |  |  |  | Risperidone | daily | 16 | 64 | 53 | 36.5 | 15.5 |
| Marder 2007c [71] | 6 | acute exacerbation of schizophrenia (DSM-IV) | oral | inpatient | Placebo | daily | 0 | 110 | 82 | 42.3 | 16 |
|  |  |  |  |  | Olanzapine | daily | 10 | 110 | 88 | 40.5 | 15.95 |
|  |  |  |  |  | Paliperidone | daily | 6 | 112 | 76 | 42.1 | 16.7 |
|  |  |  |  |  | Paliperidone | daily | 12 | 112 | 77 | 41.4 | 17 |
| Matsumoto 2018 [72] | 6 | schizophrenia (DSM-IV-TR) | oral | both | Aripiprazole | daily | 2 | 35 | 14 | 15.1 | 1.85 |
|  |  |  |  |  | Aripiprazole | daily | 8.20 | 30 | 19 | 15.4 | 2.05 |
|  |  |  |  |  | Aripiprazole | daily | 25.50 | 41 | 18 | 14.7 | 1.29 |
| McEvoy 2007b [73] | 6 | schizophrenia (DSM-IV) | oral | inpatient | Placebo | daily | 0 | 108 | 83 | 41.2 | 16.7 |
|  |  |  |  |  | Aripiprazole | daily | 10 | 106 | 82 | 40 | 16.1 |
|  |  |  |  |  | Aripiprazole | daily | 15 | 106 | 79 | 40 | 16.4 |
|  |  |  |  |  | Aripiprazole | daily | 20 | 100 | 82 | 40.4 | 16.6 |
| Meltzer 2004 [74] | 6 | acute schizophrenia or schizoaffective disorder (DSM-IV) | oral | inpatient | Placebo | daily | 0 | 98 | 74 | 37.4 | n.i. |
|  |  |  |  |  | Haloperidol | daily | 10 | 98 | 69 | 36 | n.i. |
| Meltzer 2007a [75] | 6 | acute exacerbation of schizophrenia (DSM-IV-TR) | oral | inpatient | Placebo | daily | 0 | 149 | 111 | 38.2 | n.i. |
|  |  |  |  |  | Risperidone | daily | 6 | 154 | 112 | 38.3 | n.i. |
| Meltzer 2011 [76] | 6 | acute exacerbation of schizophrenia (DSM-IV) | oral | inpatient | Placebo | daily | 0 | 114 | 88 | 37 | 12.6 |
|  |  |  |  |  | Lurasidone | daily | 40 | 119 | 93 | 37.7 | 13.3 |
|  |  |  |  |  | Lurasidone | daily | 120 | 118 | 93 | 37.9 | 14.7 |
|  |  |  |  |  | Olanzapine | daily | 15 | 122 | 95 | 38.3 | 13.2 |
| Meltzer 2012 [77] | 6 | schizophrenia (DSM-IV) | oral | both | Haloperidol | daily | 2 | 87 | 54 | 40.7 | n.i. |
|  |  |  |  |  | Risperidone | daily | 2 | 86 | 52 | 39.3 | n.i. |
|  |  |  |  |  | Risperidone | daily | 6 | 87 | 52 | 39.4 | n.i. |
| Meltzer 2014 [78] | 24 | schizophrenia or schizoaffective disorder (DSM-IV-TR) | depot | both | Risperidone | every 2 weeks | 50 | 82 | 60 | 39.2 | 17.9 |
|  |  |  |  |  | Risperidone | every 2 weeks | 100 | 78 | 56 | 41 | 20 |
| Meltzer 2015 [79] | 12 | schizophrenia (DSM-IV-TR) | depot | inpatient | Placebo | every 4 weeks | 0 | 208 | 139 | 39.5 | n.i. |
|  |  |  |  |  | Aripiprazole | every 4 weeks | 441 | 207 | 141 | 39.9 | n.i. |
|  |  |  |  |  | Aripiprazole | every 4 weeks | 882 | 208 | 143 | 39.7 | n.i. |
| Meltzer 2020 [80] | 24 | Schizophrenia or schizoaffective disorder (DSM-IV-TR) | oral | outpatient | Lurasidone | daily | 80 | 34 | 14 | 47 | 27.7 |
|  |  |  |  |  | Lurasidone | daily | 240 | 33 | 15 | 45.2 | 24.8 |
| Merlo 2000 [81] | 8 | schizophrenia, schizophreniform disorder, or schizoaffective disorder (DSM-IV) | oral | n.i. | Risperidone | daily | 2 | 23 | 14 | 23.2 | n.i. |
|  |  |  |  |  | Risperidone | daily | 4 | 26 | 13 | 26 | n.i. |
| Nakamura 2009 [82] | 6 | schizophreniform or schizoaffective disorder or catatonic or residual subtypes of schizophrenia (DSM-IV) | oral | inpatient | Placebo | daily | 0 | 90 | 70 | 41.9 | n.i. |
|  |  |  |  |  | Lurasidone | daily | 80 | 90 | 68 | 39.7 | n.i. |
| Nakamura 2016 [83] | 12 | schizophrenia (DSM-IV-TR) | oral | n.i. | Cariprazine | daily | 3 | 11 | 6 | 43.2 | 14 |
|  |  |  |  |  | Cariprazine | daily | 6 | 16 | 7 | 43.1 | 15.5 |
|  |  |  |  |  | Cariprazine | daily | 9 | 11 | 5 | 43.2 | 14.6 |
| Nasrallah 2010 [84] | 13 | schizophrenia (DSM-IV-TR) | depot | inpatient | Placebo | every 4 weeks | 0 | 127 | 78 | 41.1 | 4.2 |
|  |  |  |  |  | Paliperidone | every 4 weeks | 25 | 131 | 85 | 40.8 | 14.5 |
|  |  |  |  |  | Paliperidone | every 4 weeks | 50 | 129 | 93 | 39 | 14.5 |
|  |  |  |  |  | Paliperidone | every 4 weeks | 100 | 131 | 85 | 42.3 | 14.9 |
| Nasrallah 2013 [85] | 6 | schizophrenia (DSM-IV) | oral | inpatient | Placebo | daily | 0 | 128 | 90 | 38.2 | 14 |
|  |  |  |  |  | Lurasidone | daily | 40 | 125 | 82 | 40.7 | 16.4 |
|  |  |  |  |  | Lurasidone | daily | 80 | 123 | 76 | 38.6 | 13.5 |
|  |  |  |  |  | Lurasidone | daily | 120 | 124 | 92 | 37.7 | 13.2 |
| Nasser 2016 [86] | 8 | schizophrenia (DSM-IV-TR) | depot (subcutaneous) | inpatient | Placebo | every 4 weeks | 0 | 119 | 81 | 42.8 | 16.2 |
|  |  |  |  |  | Risperidone | every 4 weeks | 90 | 116 | 93 | 40.5 | 15 |
|  |  |  |  |  | Risperidone | every 4 weeks | 120 | 119 | 84 | 40.4 | 13.5 |
| NCT00905307 [87] | 6 | Schizophrenia (DSM-IV-TR) | oral | inpatient | Placebo | daily | 0 | 95 | 58 | 38.8 | n.i. |
|  |  |  |  |  | Brexpiprazole | daily | 0.25 | 42 | 27 | 40.4 | n.i. |
|  |  |  |  |  | Brexpiprazole | daily | 1 | 89 | 53 | 39.2 | n.i. |
|  |  |  |  |  | Brexpiprazole | daily | 2.50 | 90 | 60 | 37.4 | n.i. |
|  |  |  |  |  | Brexpiprazole | daily | 5 | 93 | 55 | 39.5 | n.i. |
|  |  |  |  |  | Aripiprazole | daily | 15 | 50 | 34 | 40.8 | n.i. |
| Ogasa 2012 [88] | 6 | schizophrenia (DSM-IV) | oral | inpatient | Placebo | daily | 0 | 50 | 42 | 38.1 | n.i. |
|  |  |  |  |  | Lurasidone | daily | 40 | 50 | 36 | 39.8 | n.i. |
|  |  |  |  |  | Lurasidone | daily | 120 | 49 | 36 | 41 | n.i. |
| Oosthuizen 2004 [89] | 6 | schizophreniform disorder, schizophrenia or schizoaﬀective disorder and a life-time neuroleptic exposure of 4 wk or less (DSM-IV) | oral | both | Haloperidol | daily | 2 | 20 | 14 | 26.8 | n.i. |
|  |  |  |  |  | Haloperidol | daily | 8 | 20 | 10 | 28.9 | n.i. |
| Pandina 2010 [90] | 13 | schizophrenia (DSM-IV) | depot | inpatient | Placebo | every 4 weeks | 0 | 164 | 109 | 39 | n.i. |
|  |  |  |  |  | Paliperidone | every 4 weeks | 25 | 160 | 116 | 39 | n.i. |
|  |  |  |  |  | Paliperidone | every 4 weeks | 100 | 165 | 110 | 39 | n.i. |
|  |  |  |  |  | Paliperidone | every 4 weeks | 150 | 163 | 105 | 39 | n.i. |
| Patil 2007 [91] | 4 | schizophrenia (DSM-IV-TR) | oral | inpatient | Placebo | daily | 0 | 63 | 49 | 41 | n.i. |
|  |  |  |  |  | Olanzapine | daily | 15 | 34 | 25 | 42.3 | n.i. |
| Peuskens 1995 [92] | 8 | schizophrenia (DSM-III-R) | oral | inpatient | Haloperidol | daily | 10 | 226 | 150 | 38.1 | n.i. |
|  |  |  |  |  | Risperidone | daily | 1 | 229 | 166 | 38.4 | n.i. |
|  |  |  |  |  | Risperidone | daily | 4 | 227 | 152 | 38.1 | n.i. |
|  |  |  |  |  | Risperidone | daily | 8 | 230 | 144 | 37.6 | n.i. |
|  |  |  |  |  | Risperidone | daily | 12 | 226 | 142 | 37.9 | n.i. |
|  |  |  |  |  | Risperidone | daily | 16 | 224 | 150 | 38.5 | n.i. |
| Potkin 2003 [93] | 4 | schizophrenia or schizoaffective disorder (DSM-IV) | oral | inpatient | Aripiprazole | daily | 20 | 101 | 73 | 38.1 | n.i. |
|  |  |  |  |  | Aripiprazole | daily | 30 | 101 | 66 | 40.2 | n.i. |
|  |  |  |  |  | Placebo | daily | 0 | 103 | 73 | 38.8 | n.i. |
|  |  |  |  |  | Risperidone | daily | 6 | 99 | 71 | 38.6 | n.i. |
| Potkin 2007c [94] | 6 | acute exacerbation of schizophrenia (DSM-IV) | oral | inpatient | Asenapine | daily | 10 | 60 | 46 | 38 | n.i. |
|  |  |  |  |  | Placebo | daily | 0 | 62 | 49 | 42 | n.i. |
|  |  |  |  |  | Risperidone | daily | 6 | 60 | 36 | 43 | n.i. |
| Potkin 2008a [95] | 6 | acute exacerbation of schizophrenia (DSM-IV) | oral | outpatient | Haloperidol | daily | 15 | 124 | 86 | 39.1 | 15.6 |
|  |  |  |  |  | Placebo | daily | 0 | 127 | 90 | 39.3 | 15.6 |
|  |  |  |  |  | Iloperidone | daily | 4 | 121 | 82 | 38.4 | n.i. |
|  |  |  |  |  | Iloperidone | daily | 8 | 125 | 94 | 37 | n.i. |
|  |  |  |  |  | Iloperidone | daily | 12 | 124 | 91 | 40.1 | 15.6 |
| Potkin 2008b [96] | 6 | schizophrenia and schizoaffective disorder (DSM-IV) | oral | inpatient | Iloperidone | daily | 7.16 | 153 | 106 | 38.4 | n.i. |
|  |  |  |  |  | Iloperidone | daily | 14.58 | 154 | 109 | 39.3 | n.i. |
|  |  |  |  |  | Placebo | daily | 0 | 156 | 105 | 38.8 | n.i. |
|  |  |  |  |  | Risperidone | daily | 7.02 | 153 | 115 | 37.5 | n.i. |
| Potkin 2008c [97] | 6 | acute exacerbation of schizophrenia and schizoaffective disorder (DSM-IV) | oral | inpatient | Iloperidone | daily | 14.42 | 244 | 146 | 38.9 | n.i. |
|  |  |  |  |  | Iloperidone | daily | 22.17 | 145 | 99 | 37.3 | n.i. |
|  |  |  |  |  | Placebo | daily | 0 | 160 | 94 | 39 | 15.6 |
|  |  |  |  |  | Risperidone | daily | 7.09 | 157 | 96 | 39.8 | 14.4 |
| Potkin 2015 [98] | 6 | acute exacerbation of schizophrenia (DSM-IV) | oral | inpatient | Placebo | daily | 0 | 72 | 55 | 41 | n.i. |
|  |  |  |  |  | Haloperidol | daily | 10 | 73 | 58 | 40 | n.i. |
|  |  |  |  |  | Lurasidone | daily | 20 | 71 | 51 | 40.7 | n.i. |
|  |  |  |  |  | Lurasidone | daily | 40 | 69 | 46 | 42 | n.i. |
|  |  |  |  |  | Lurasidone | daily | 80 | 71 | 52 | 42.2 | n.i. |
| Puech 1998 [99] | 4 | chronic or subchronic schizophrenia with acute exacerbation (DSM-III-R) | oral | inpatient | Amisulpride | daily | 100 | 61 | 42 | 36.4 | 11.6 |
|  |  |  |  |  | Amisulpride | daily | 400 | 64 | 43 | 35.6 | 9.7 |
|  |  |  |  |  | Amisulpride | daily | 800 | 65 | 31 | 36.7 | 9.6 |
|  |  |  |  |  | Amisulpride | daily | 1200 | 65 | 45 | 34.8 | 9.3 |
|  |  |  |  |  | Haloperidol | daily | 16 | 64 | 36 | 36.6 | 10.7 |
| Saito 2020 [100] | 6 | schizophrenia (DSM-IV-TR) | oral | both | Placebo | daily | 0 | 47 | 20 | 15.6 | 2.13 |
|  |  |  |  |  | Blonanserin | daily | 8 | 51 | 21 | 15.3 | 1.98 |
|  |  |  |  |  | Blonanserin | daily | 16 | 53 | 23 | 15.6 | 2.53 |
| Schmidt 2014 [101] | 6 | schizophrenia, acute schizophrenia (DSM-IV) | oral | inpatient | Placebo | daily | 0 | 101 | 59 | 38 | 10.9 |
|  |  |  |  |  | Olanzapine | daily | 15 | 93 | 49 | 38.6 | 10.7 |
| Shen 2014 [102] | 6 | acute schizophrenia (DSM-IV-TR) | oral | inpatient | Placebo | daily | 0 | 78 | 52 | 39.6 | n.i. |
|  |  |  |  |  | Olanzapine | daily | 15 | 77 | 46 | 40.1 | n.i. |
| Singh 2011 [103] | 6 | Schizophrenia (DSM-IV) | oral | inpatient | Placebo | daily | 0 | 51 | 23 | 15.7 | 2.3 |
|  |  |  |  |  | Paliperidone | daily | 1.50 | 54 | 30 | 15.1 | 2.6 |
|  |  |  |  |  | Paliperidone | daily | 5 | 48 | 31 | 15.3 | 2.3 |
|  |  |  |  |  | Paliperidone | daily | 10.34 | 47 | 33 | 15.5 | 2.7 |
| Study 115 2000 [104] | 6 | chronic or subchronic (less than one year) schizophrenia with acute exacerbation, or schizoaffective disorder (DSM-III-R) | oral | inpatient | Haloperidol | daily | 15 | 85 | 60 | 38.8 | n.i. |
|  |  |  |  |  | Placebo | daily | 0 | 83 | 54 | 38.9 | n.i. |
|  |  |  |  |  | Ziprasidone | daily | 40 | 87 | 53 | 40.3 | n.i. |
|  |  |  |  |  | Ziprasidone | daily | 120 | 78 | 55 | 40 | n.i. |
|  |  |  |  |  | Ziprasidone | daily | 200 | 86 | 55 | 38.2 | n.i. |
| Study 93202 2002 [105] | 4 | schizophrenia acute relapse (DSM-III-R) | oral | inpatient | Aripiprazole | daily | 30 | 34 | 32 | 33 | n.i. |
|  |  |  |  |  | Haloperidol | daily | 20 | 34 | 30 | 38.8 | n.i. |
|  |  |  |  |  | Placebo | daily | 0 | 35 | 29 | 37.9 | n.i. |
| Study 94202 2002 [105] | 4 | schizophrenia (DSM-IV) | oral | inpatient | Placebo | daily | 0 | 64 | 53 | 38.02 | n.i. |
|  |  |  |  |  | Aripiprazole | daily | 2 | 59 | 47 | 39.84 | n.i. |
|  |  |  |  |  | Aripiprazole | daily | 10 | 60 | 49 | 37.8 | n.i. |
|  |  |  |  |  | Aripiprazole | daily | 30 | 61 | 46 | 38.8 | n.i. |
|  |  |  |  |  | Haloperidol | daily | 10 | 63 | 52 | 38.91 | n.i. |
| Study RIS-USA-72 1996 [106] | 4 | schizophrenia (DSM-IV) | oral | inpatient | Placebo | daily | 0 | 83 | 65 | 37 | n.i. |
|  |  |  |  |  | Risperidone | daily | 4 | 85 | 67 | 38 | n.i. |
|  |  |  |  |  | Risperidone | daily | 8 | 78 | 64 | 38.5 | n.i. |
| Takahashi 2013 [107] | 13 | schizophrenia (DSM-IV-TR) | depot | n.i. | Placebo | every 4 weeks | 0 | 164 | 83 | 44 | 16.5 |
|  |  |  |  |  | Paliperidone | every 4 weeks | 75 | 160 | 101 | 46 | 18.6 |
| van Kammen 1996 [108] | 5.7 | schizophrenia (DSM-III-R) | oral | inpatient | Placebo | daily | 0 | 48 | 45 | 37.1 | 13.8 |
|  |  |  |  |  | Sertindole | daily | 8 | 52 | 33 | 39.7 | 16.6 |
|  |  |  |  |  | Sertindole | daily | 12 | 51 | 38 | 38.6 | 14.4 |
|  |  |  |  |  | Sertindole | daily | 20 | 54 | 39 | 36.8 | 12.2 |
| Walling 2019 [109] | 4 | schizophrenia (DSM-IV-TR) | oral | inpatient | Placebo | daily | 0 | 74 | 56 | 41.2 | 19.6 |
|  |  |  |  |  | Risperidone | daily | 6 | 37 | 25 | 41.3 | 18 |
| Zborowski 1995 [110] | 8 | schizophrenia (DSM-III-R/DSM-IV) | oral | inpatient | Haloperidol | daily | 16 | 115 | 86 | 39 | 15.4 |
|  |  |  |  |  | Placebo | daily | 0 | 116 | 90 | 38 | 15.8 |
|  |  |  |  |  | Sertindole | daily | 20 | 117 | 89 | 38 | 15.7 |
|  |  |  |  |  | Sertindole | daily | 24 | 113 | 85 | 37 | 13.8 |
| Zimbroff 1997 [111] | 8 | schizophrenia (DSM-III-R/DSM-IV) | oral | inpatient | Placebo | daily | 0 | 73 | 57 | 38.7 | 16.6 |
|  |  |  |  |  | Haloperidol | daily | 4 | 71 | 59 | 38.1 | 14.9 |
|  |  |  |  |  | Haloperidol | daily | 8 | 67 | 54 | 39.9 | 16.7 |
|  |  |  |  |  | Haloperidol | daily | 16 | 70 | 53 | 39 | 18.6 |
|  |  |  |  |  | Sertindole | daily | 12 | 76 | 61 | 37.8 | 15.4 |
|  |  |  |  |  | Sertindole | daily | 20 | 68 | 52 | 40.4 | 17.3 |
|  |  |  |  |  | Sertindole | daily | 24 | 72 | 50 | 39.5 | 17 |

N= number of participants randomised, ICD 9/10 = International Classification of Diseases, 9th/10th Revision, DSM-III, -III-R, -IV, -IV-TR, -V = different versions of the Diagnostic and Statistical Manual of Mental Disorders, n.i. = not indicated, IR= immediate release, XR= extended release. Some reports provided data for several studies.

a: study only reported positive symptoms, b: studies only reported negative symptoms,

**References**

1.	Arvanitis, L.A. and B.G. Miller, *Multiple fixed doses of “Seroquel” (quetiapine) in patients with acute exacerbation of schizophrenia: A comparison with haloperidol and placebo.* Biological Psychiatry, 1997. **42**(4): p. 233-246.

2.	Beasley, C.M., et al., *Olanzapine versus placebo: results of a double-blind, fixed-dose olanzapine trial.* Psychopharmacology, 1996. **124**(1-2): p. 159-167.

3.	Beasley, C.M., et al., *Olanzapine versus Placebo and Haloperidol.* Neuropsychopharmacology, 1996. **14**(2): p. 111-123.

4.	Beasley, C.M., et al., *Olanzapine versus haloperidol: acute phase results of the international double-blind olanzapine trial.* European Neuropsychopharmacology, 1997. **7**(2): p. 125-137.

5.	Berger, G.E., et al., *Dosing quetiapine in drug-naive first-episode psychosis: a controlled, double-blind, randomized, single-center study investigating efficacy, tolerability, and safety of 200 mg/day vs. 400 mg/day of quetiapine fumarate in 141 patients aged 15 to 25 years.* J Clin Psychiatry, 2008. **69**(11): p. 1702-14.

6.	Boyer, P., et al., *Treatment of Negative Symptoms in Schizophrenia with Amisulpride.* British Journal of Psychiatry, 1995. **166**(1): p. 68-72.

7.	Bugarski-Kirola, D., et al., *A phase II/III trial of bitopertin monotherapy compared with placebo in patients with an acute exacerbation of schizophrenia - results from the CandleLyte study.* Eur Neuropsychopharmacol, 2014. **24**(7): p. 1024-36.

8.	Cantillon, M., *EFFICACY AND SAFETY OF NOVEL DOPAMINE SEROTONIN STABILIZER RP 5063 IN ACUTE SCHIZOPHRENIA AND SCHIZOAFFECTIVE DISORDER.* Schizophrenia Research, 2014. **153**: p. S22.

9.	Canuso, C.M., et al., *A Randomized, Double-Blind, Placebo-Controlled Study of 2 Dose Ranges of Paliperidone Extended-Release in the Treatment of Subjects With Schizoaffective Disorder.* The Journal of Clinical Psychiatry, 2010. **71**(05): p. 587-598.

10.	Casey, D.E., et al., *Efficacy and safety of bifeprunox in patients with an acute exacerbation of schizophrenia: results from a randomized, double-blind, placebo-controlled, multicenter, dose-finding study.* Psychopharmacology, 2008. **200**(3): p. 317-331.

11.	Chouinard, G., et al., *A CANADIAN MULTICENTER PLACEBO-CONTROLLED STUDY OF FIXED DOSES OF RISPERIDONE AND HALOPERIDOL IN THE TREATMENT OF CHRONIC-SCHIZOPHRENIC PATIENTS.* Journal of Clinical Psychopharmacology, 1993. **13**(1): p. 25-40.

12.	Citrome, L., et al., *Efficacy and Safety of HP-3070, an Asenapine Transdermal System, in Patients With Schizophrenia: A Phase 3, Randomized, Placebo-Controlled Study.* Journal of Clinical Psychiatry, 2021. **82**(1).

13.	Cooper, S.J., et al., *A placebo-controlled comparison of zotepine versus chlorpromazine in patients with acute exacerbation of schizophrenia.* Acta Psychiatrica Scandinavica, 2000. **101**(3): p. 218-225.

14.	Correll, C.U., et al., *Efficacy and Safety of Brexpiprazole for the Treatment of Acute Schizophrenia: A 6-Week Randomized, Double-Blind, Placebo-Controlled Trial.* Am J Psychiatry, 2015. **172**(9): p. 870-80.

15.	Correll, C.U., et al., *Efficacy and Safety of Lumateperone for Treatment of Schizophrenia.* JAMA Psychiatry, 2020a. **77**(4): p. 349.

16.	Correll, C.U., et al., *Efficacy and safety of once-monthly Risperidone ISM® in schizophrenic patients with an acute exacerbation.* npj Schizophrenia, 2020b. **6**(1).

17.	Corrigan, M.H., et al., *Effectiveness of the selective D4 antagonist sonepiprazole in schizophrenia: a placebo-controlled trial.* Biological Psychiatry, 2004. **55**(5): p. 445-451.

18.	Cutler, A.J., et al., *The Efficacy and Safety of Lower Doses of Aripiprazole for the Treatment of Patients with Acute Exacerbation of Schizophrenia.* CNS Spectrums, 2006. **11**(9): p. 691-702.

19.	Cutler, A.J., et al., *Four-Week, Double-Blind, Placebo- and Ziprasidone-Controlled Trial of Iloperidone in Patients With Acute Exacerbations of Schizophrenia.* Journal of Clinical Psychopharmacology, 2008. **28**(2): p. S20-S28.

20.	Cutler, A.J., et al., *A Failed 6-Week, Randomized, Double-Blind, Placebo-Controlled Study of Once-daily Extended Release Quetiapine Fumarate in Patients with Acute Schizophrenia: Lessons Learned.* Psychopharmacology Bulletin, 2010. **43**(4): p. 37-69.

21.	Daniel, D., *Ziprasidone 80 mg/day and 160 mg/day in the Acute Exacerbation of Schizophrenia and Schizoaffective Disorder A 6-Week Placebo-Controlled Trial.* Neuropsychopharmacology, 1999. **20**(5): p. 491-505.

22.	Danion, J.-M., et al., *Improvement of Schizophrenic Patients With Primary Negative Symptoms Treated With Amisulpride.* American Journal of Psychiatry, 1999. **156**(4): p. 610-616.

23.	Davidson, M., et al., *Efficacy, safety and early response of paliperidone extended-release tablets (paliperidone ER): Results of a 6-week, randomized, placebo-controlled study.* Schizophrenia Research, 2007. **93**(1-3): p. 117-130.

24.	Durgam, S., et al., *An evaluation of the safety and efficacy of cariprazine in patients with acute exacerbation of schizophrenia: A phase II, randomized clinical trial.* Schizophrenia Research, 2014. **152**(2-3): p. 450-457.

25.	Durgam, S., et al., *Cariprazine in Acute Exacerbation of Schizophrenia.* The Journal of Clinical Psychiatry, 2015. **76**(12): p. e1574-e1582.

26.	Durgam, S., et al., *Cariprazine in the treatment of schizophrenia.* International Clinical Psychopharmacology, 2016. **31**(2): p. 61-68.

27.	Egan, M.F., et al., *Randomized controlled study of the T‐type calcium channel antagonist MK‐8998 for the treatment of acute psychosis in patients with schizophrenia.* Human Psychopharmacology: Clinical and Experimental, 2013. **28**(2): p. 124-133.

28.	Fabre, L.F., et al., *ICI 204,636, a novel, atypical antipsychotic: early indication of safety and efficacy in patients with chronic and subchronic schizophrenia.* Clinical Therapeutics, 1995. **17**(3): p. 366-378.

29.	Findling, R.L., et al., *A multiple-center, randomized, double-blind, placebo-controlled study of oral aripiprazole for treatment of adolescents with schizophrenia.* Am J Psychiatry, 2008. **165**(11): p. 1432-41.

30.	Findling, R.L., et al., *Efficacy and safety of quetiapine in adolescents with schizophrenia investigated in a 6-week, double-blind, placebo-controlled trial.* J Child Adolesc Psychopharmacol, 2012. **22**(5): p. 327-42.

31.	Findling, R.L., et al., *Safety and Efficacy from an 8 Week Double-Blind Trial and a 26 Week Open-Label Extension of Asenapine in Adolescents with Schizophrenia.* J Child Adolesc Psychopharmacol, 2015. **25**(5): p. 384-96.

32.	Garcia, E., et al., *The efficacy and safety of blonanserin compared with haloperidol in acute-phase schizophrenia: a randomized, double-blind, placebo-controlled, multicentre study.* CNS Drugs, 2009. **23**(7): p. 615-25.

33.	Goff, D.C., et al., *An Exploratory Haloperidol-Controlled Dose-Finding Study of Ziprasidone in Hospitalized Patients With Schizophrenia or Schizoaffective Disorder.* Journal of Clinical Psychopharmacology, 1998. **18**(4): p. 296-304.

34.	Goff, D.C., et al., *High-Dose Oral Ziprasidone Versus Conventional Dosing in Schizophrenia Patients With Residual Symptoms.* Journal of Clinical Psychopharmacology, 2013. **33**(4): p. 485-490.

35.	Goldman, R., et al., *Efficacy and Safety of Lurasidone in Adolescents with Schizophrenia: A 6-Week, Randomized Placebo-Controlled Study.* J Child Adolesc Psychopharmacol, 2017. **27**(6): p. 516-525.

36.	Gopal, S., et al., *Efficacy and safety of paliperidone palmitate in adult patients with acutely symptomatic schizophrenia: a randomized, double-blind, placebo-controlled, dose-response study.* International Clinical Psychopharmacology, 2010. **25**(5): p. 247-256.

37.	Hale, J.-M.A.S.A., *Sertindole improves both the positive and negative symptoms of schizophrenia: Results of a phase III trial.* International Journal of Psychiatry in Clinical Practice, 2000. **4**(1): p. 55-62.

38.	Haas, M., et al., *Efficacy, safety and tolerability of two dosing regimens in adolescent schizophrenia: double-blind study.* Br J Psychiatry, 2009. **194**(2): p. 158-64.

39.	Higuchi, T., et al., *Lurasidone in the treatment of schizophrenia: Results of a double‐blind, placebo‐controlled trial in Asian patients.* Asia-Pacific Psychiatry, 2019. **11**(2).

40.	Higuchi, T., et al., *Randomized, double‐blind, placebo, and risperidone‐controlled study of lurasidone in the treatment of schizophrenia: Results of an inconclusive 6‐week trial.* Asia-Pacific Psychiatry, 2019. **11**(3).

41.	*Hirayasu Y, Tomioka M, Iizumi M, Kikuchi H (2010): A double-blind, placebo-controlled, comparative study of paliperidone extended release (ER) tablets in patients with schizophrenia. Jpn J Clin Psychopharmacol. 13:2077-2103.*

42.	Honer, W.G., et al., *A Randomized, Double-Blind, Placebo-Controlled Study of the Safety and Tolerability of High-Dose Quetiapine in Patients With Persistent Symptoms of Schizophrenia or Schizoaffective Disorder.* Journal of Clinical Psychiatry, 2012. **73**(1): p. 13-20.

43.	Ishigooka, J., S. Iwashita, and Y. Tadori, *Efficacy and safety of brexpiprazole for the treatment of acute schizophrenia in Japan: A 6-week, randomized, double-blind, placebo-controlled study.* Psychiatry and Clinical Neurosciences, 2018. **72**(9): p. 692-700.

44.	Iwata, N., et al., *Efficacy and safety of blonanserin transdermal patch in patients with schizophrenia: A 6-week randomized, double-blind, placebo-controlled, multicenter study.* Schizophr Res, 2020. **215**: p. 408-415.

45.	Iyo, M., et al., *Efficacy and safety of lurasidone in acutely psychotic patients with schizophrenia: A 6‐week, randomized, double‐blind, placebo‐controlled study.* Psychiatry and Clinical Neurosciences, 2021. **75**(7): p. 227-235.

46.	Kahn, R.S., et al., *Efficacy and tolerability of once-daily extended release quetiapine fumarate in acute schizophrenia: a randomized, double-blind, placebo-controlled study.* J Clin Psychiatry, 2007. **68**(6): p. 832-42.

47.	Kane, J.M., et al., *Efficacy and Safety of Aripiprazole and Haloperidol Versus Placebo in Patients With Schizophrenia and Schizoaffective Disorder.* The Journal of Clinical Psychiatry, 2002. **63**(9): p. 763-771.

48.	Kane, J.M., et al., *Long-Acting Injectable Risperidone: Efficacy and Safety of the First Long-Acting Atypical Antipsychotic.* American Journal of Psychiatry, 2003. **160**(6): p. 1125-1132.

49.	Kane, J., et al., *Treatment of schizophrenia with paliperidone extended-release tablets: a 6-week placebo-controlled trial.* Schizophr Res, 2007. **90**(1-3): p. 147-61.

50.	Kane, J.M., et al., *Efficacy and Safety of Asenapine in a Placebo- and Haloperidol-Controlled Trial in Patients With Acute Exacerbation of Schizophrenia.* Journal of Clinical Psychopharmacology, 2010. **30**(2): p. 106-115.

51.	Kane, J.M., et al., *Aripiprazole Once-Monthly in the Acute Treatment of Schizophrenia: Findings From a 12-Week, Randomized, Double-Blind, Placebo-Controlled Study.* Journal of Clinical Psychiatry, 2014. **75**(11): p. 1254-1260.

52.	Kane, J.M., et al., *A multicenter, randomized, double-blind, controlled phase 3 trial of fixed-dose brexpiprazole for the treatment of adults with acute schizophrenia.* Schizophr Res, 2015. **164**(1-3): p. 127-35.

53.	Kane, J.M., et al., *Efficacy and Safety of Cariprazine in Acute Exacerbation of Schizophrenia.* Journal of Clinical Psychopharmacology, 2015. **35**(4): p. 367-373.

54.	Keck Jr, P., et al., *Ziprasidone 40 and 120 mg/day in the acute exacerbation of schizophrenia and schizoaffective disorder: a 4-week placebo-controlled trial.* Psychopharmacology, 1998. **140**(2): p. 173-184.

55.	King, D.J., C.G.G. Link, and B. Kowalcyk, *A comparison of bd and tid dose regimens of quetiapine (Seroquel) in the treatment of schizophrenia.* Psychopharmacology, 1998. **137**(2): p. 139-146.

56.	Kinon, B.J., et al., *Standard and Higher Dose of Olanzapine in Patients With Schizophrenia or Schizoaffective Disorder.* Journal of Clinical Psychopharmacology, 2008. **28**(4): p. 392-400.

57.	Kinon, B.J., et al., *A Multicenter, Inpatient, Phase 2, Double-Blind, Placebo-Controlled Dose-Ranging Study of LY2140023 Monohydrate in Patients With DSM-IV Schizophrenia.* Journal of Clinical Psychopharmacology, 2011. **31**(3): p. 349-355.

58.	Kinoshita, T., et al., *Efficacy and safety of asenapine in Asian patients with an acute exacerbation of schizophrenia: a multicentre, randomized, double-blind, 6-week, placebo-controlled study.* Psychopharmacology (Berl), 2016. **233**(14): p. 2663-74.

59.	Kramer, M., et al., *Paliperidone palmitate, a potential long-acting treatment for patients with schizophrenia. Results of a randomized, double-blind, placebo-controlled efficacy and safety study.* The International Journal of Neuropsychopharmacology, 2009. **13**(05): p. 635-647.

60.	Landbloom, R., et al., *Asenapine for the treatment of adults with an acute exacerbation of schizophrenia: results from a randomized, double-blind, fixed-dose, placebo-controlled trial with olanzapine as an active control.* CNS Spectrums, 2016. **22**(4): p. 333-341.

61.	Lauriello, J., et al., *An 8-Week, Double-Blind, Randomized, Placebo-Controlled Study of Olanzapine Long-Acting Injection in Acutely Ill Patients With Schizophrenia.* The Journal of Clinical Psychiatry, 2008. **69**(5): p. 790-799.

62.	Lecrubier, Y., et al., *The treatment of negative symptoms and deficit states of chronic schizophrenia: olanzapine compared to amisulpride and placebo in a 6‐month double‐blind controlled clinical trial.* Acta Psychiatrica Scandinavica, 2006. **114**(5): p. 319-327.

63.	Lieberman, J.A., et al., *ITI-007 for the Treatment of Schizophrenia: A 4-Week Randomized, Double-Blind, Controlled Trial.* Biological Psychiatry, 2016. **79**(12): p. 952-961.

64.	Lindenmayer, J.P., et al., *The efficacy and tolerability of once-daily extended release quetiapine fumarate in hospitalized patients with acute schizophrenia: a 6-week randomized, double-blind, placebo-controlled study.* Psychopharmacol Bull, 2008. **41**(3): p. 11-35.

65.	Lindenmayer, J.-P., et al., *A Randomized, Double-Blind, Parallel-Group, Fixed-Dose, Clinical Trial of Quetiapine at 600 Versus 1200 mg/d for Patients With Treatment-Resistant Schizophrenia or Schizoaffective Disorder.* Journal of Clinical Psychopharmacology, 2011. **31**(2): p. 160-168.

66.	Litman, R.E., et al., *AZD8529, a positive allosteric modulator at the mGluR2 receptor, does not improve symptoms in schizophrenia: A proof of principle study.* Schizophrenia Research, 2016. **172**(1-3): p. 152-157.

67.	Litman, R.E., et al., *The Selective Neurokinin 3 Antagonist AZD2624 Does Not Improve Symptoms or Cognition in Schizophrenia.* Journal of Clinical Psychopharmacology, 2014. **34**(2): p. 199-204.

68.	Loebel, A., et al., *Efficacy and safety of lurasidone 80 mg/day and 160 mg/day in the treatment of schizophrenia: A randomized, double-blind, placebo- and active-controlled trial.* Schizophrenia Research, 2013. **145**(1-3): p. 101-109.

69.	Loo, H., et al., *Amisulpride versus placebo in the medium-term treatment of the negative symptoms of schizophrenia.* British Journal of Psychiatry, 1997. **170**(1): p. 18-22.

70.	*Marder SR, Meibach RC (1994): Risperidone in the treatment of schizophrenia. AmJPsychiatry. 151:825-835.*

71.	Marder, S.R., et al., *Efficacy and Safety of Paliperidone Extended-Release Tablets: Results of a 6-Week, Randomized, Placebo-Controlled Study.* Biological Psychiatry, 2007. **62**(12): p. 1363-1370.

72.	Matsumoto, H., et al., *Safety and efficacy from a 6-week double-blind study and a 52-week open-label extension of aripiprazole in adolescents with schizophrenia in Japan.* Psychiatry Clin Neurosci, 2018. **72**(9): p. 701-712.

73.	McEvoy, J.P., et al., *A randomized, double-blind, placebo-controlled, study of the efficacy and safety of aripiprazole 10, 15 or 20mg/day for the treatment of patients with acute exacerbations of schizophrenia.* Journal of Psychiatric Research, 2007. **41**(11): p. 895-905.

74.	Meltzer, H.Y., et al., *Placebo-Controlled Evaluation of Four Novel Compounds for the Treatment of Schizophrenia and Schizoaffective Disorder.* American Journal of Psychiatry, 2004. **161**(6): p. 975-984.

75.	Meltzer H, Barbato L, Heisterberg J, Yeung P, Shapira N, editors. A randomized, double-blind, placebo-controlled efficacy and safety study of bifeprunox as treatment for patients with acutely exacerbated schizophrenia. Schizophrenia bulletin; 2007: OXFORD UNIV PRESS GREAT CLARENDON ST, OXFORD OX2 6DP, ENGLAND.

76.	Meltzer, H.Y., et al., *Lurasidone in the Treatment of Schizophrenia: A Randomized, Double-Blind, Placebo- and Olanzapine-Controlled Study.* American Journal of Psychiatry, 2011. **168**(9): p. 957-967.

77.	Meltzer, H.Y., et al., *Pimavanserin, a selective serotonin (5-HT)2A-inverse agonist, enhances the efficacy and safety of risperidone, 2mg/day, but does not enhance efficacy of haloperidol, 2mg/day: comparison with reference dose risperidone, 6mg/day.* Schizophr Res, 2012. **141**(2-3): p. 144-52.

78.	Meltzer, H.Y., et al., *A six month randomized controlled trial of long acting injectable risperidone 50 and 100 mg in treatment resistant schizophrenia.* Schizophrenia Research, 2014. **154**(1-3): p. 14-22.

79.	Meltzer, H.Y., et al., *A Randomized, Double-Blind, Placebo-Controlled Trial of Aripiprazole Lauroxil in Acute Exacerbation of Schizophrenia.* The Journal of Clinical Psychiatry, 2015. **76**(08): p. 1085-1090.

80.	Meltzer, H.Y., et al., *Lurasidone Improves Psychopathology and Cognition in Treatment-Resistant Schizophrenia.* Journal of Clinical Psychopharmacology, 2020. **40**(3): p. 240-249.

81.	Merlo, M.C.G., et al., *Risperidone, 2 mg/day vs. 4 mg/day, in First-Episode, Acutely Psychotic Patients.* The Journal of Clinical Psychiatry, 2002. **63**(10): p. 885-891.

82.	Nakamura, M., et al., *Lurasidone in the Treatment of Acute Schizophrenia.* The Journal of Clinical Psychiatry, 2009. **70**(6): p. 829-836.

83.	Nakamura, T., et al., *Clinical pharmacology study of cariprazine (MP-214) in patients with schizophrenia (12-week treatment).* Drug Design, Development and Therapy, 2016: p. 327.

84.	Nasrallah, H.A., et al., *A Controlled, Evidence-Based Trial of Paliperidone Palmitate, A Long-Acting Injectable Antipsychotic, in Schizophrenia.* Neuropsychopharmacology, 2010. **35**(10): p. 2072-2082.

85.	Nasrallah, H.A., et al., *Lurasidone for the treatment of acutely psychotic patients with schizophrenia: A 6-week, randomized, placebo-controlled study.* Journal of Psychiatric Research, 2013. **47**(5): p. 670-677.

86.	Nasser, A.F., et al., *Efficacy, Safety, and Tolerability of RBP-7000 Once-Monthly Risperidone for the Treatment of Acute Schizophrenia.* Journal of Clinical Psychopharmacology, 2016. **36**(2): p. 130-140.

87.	NCT00905307. Study to Evaluate the Efficacy, Safety, and Tolerability of Oral OPC-34712 and Aripiprazole for Treatment of Acute Schizophrenia (STEP 203). [https://clinicaltrials.gov/study/NCT00905307.](%20https://clinicaltrials.gov/study/NCT00905307.)

88.	Ogasa, M., et al., *Lurasidone in the treatment of schizophrenia: a 6-week, placebo-controlled study.* Psychopharmacology, 2012. **225**(3): p. 519-530.

89.	Oosthuizen, P., et al., *A randomized, controlled comparison of the efficacy and tolerability of low and high doses of haloperidol in the treatment of first-episode psychosis.* International Journal of Neuropsychopharmacology, 2004. **7**(2): p. 125-131.

90.	Pandina, G.J., et al., *A Randomized, Placebo-Controlled Study to Assess the Efficacy and Safety of 3 Doses of Paliperidone Palmitate in Adults With Acutely Exacerbated Schizophrenia.* Journal of Clinical Psychopharmacology, 2010. **30**(3): p. 235-244.

91.	Patil, S.T., et al., *Activation of mGlu2/3 receptors as a new approach to treat schizophrenia: a randomized Phase 2 clinical trial.* Nature Medicine, 2007. **13**(9): p. 1102-1107.

92.	Peuskens, J., *Risperidone in the Treatment of Patients with Chronic Schizophrenia: a Multi-National, Multi-Centre, Double-Blind, Parallel-Group Study versus Haloperidol.* British Journal of Psychiatry, 1995. **166**(6): p. 712-726.

93.	Potkin, S.G., et al., *Aripiprazole, an Antipsychotic With a Novel Mechanism of Action, and Risperidone vs Placebo in Patients With Schizophrenia and Schizoaffective Disorder.* Archives of General Psychiatry, 2003. **60**(7): p. 681.

94.	Potkin, S.G. and M. Cohen, *Efficacy and Tolerability of Asenapine in Acute Schizophrenia.* The Journal of Clinical Psychiatry, 2007. **68**(10): p. 1492-1500.

95.	Cucchiaro, J.B., et al., *Risk/benefit assessment of iloperidone in the treatment of psychotic disorders: A prospective double-blind, randomized, parallel-group, placebo-controlled study.* European Neuropsychopharmacology, 2001. **11**: p. S292-S293.

96.	Potkin, S.G., et al., *Efficacy of Iloperidone in the Treatment of Schizophrenia.* Journal of Clinical Psychopharmacology, 2008. **28**(2): p. S4-S11.

97.	Potkin, S.G., et al., *Efficacy of iloperidone in the treatment of schizophrenia: initial phase 3 studies.* J Clin Psychopharmacol, 2008. **28**(2 Suppl 1): p. S4-11.

98.	Potkin, S.G., T. Kimura, and J. Guarino, *A 6-week, double-blind, placebo- and haloperidol-controlled, phase II study of lurasidone in patients with acute schizophrenia.* Therapeutic Advances in Psychopharmacology, 2015. **5**(6): p. 322-331.

99.	Puech, A., O. Fleurot, and W. Rein, *Amisulpride, an atypical antipsychotic, in the treatment of acute episodes of schizophrenia: a dose‐ranging study vs. haloperidol.* Acta Psychiatrica Scandinavica, 1998. **98**(1): p. 65-72.

100.	Saito, T., et al., *Efficacy and Safety of Blonanserin Oral Tablet in Adolescents with Schizophrenia: A 6-Week, Randomized Placebo-Controlled Study.* J Child Adolesc Psychopharmacol, 2022. **32**(1): p. 12-23.

101.	Schmidt, M.E., et al., *A double-blind, randomized, placebo-controlled study with JNJ-37822681, a novel, highly selective, fast dissociating D2 receptor antagonist in the treatment of acute exacerbation of schizophrenia.* European Neuropsychopharmacology, 2012. **22**(10): p. 721-733.

102.	Shen, J.H., et al., *A 6-week randomized, double-blind, placebo-controlled, comparator referenced trial of vabicaserin in acute schizophrenia.* J Psychiatr Res, 2014. **53**: p. 14-22.

103.	Singh, J., et al., *A randomized, double-blind study of paliperidone extended-release in treatment of acute schizophrenia in adolescents.* Biol Psychiatry, 2011. **70**(12): p. 1179-87.

104.	*115 2000 S (2000): Center for drug evaluation and research approval package for application number 20-825. Medical review. http://www.fda.gov.*

105.	*2002 S9 (2002): Center for drug evaluation and research. Application number 21-436. Medical review(s). http://www.fda.gov.*

106.	*1996 SR-U-7 (1996): Office of Clinical pharmacology and biopharmacy review. NDA number: 20272. Janssen-Cilag, data on file.*

107.	Takahashi, N., et al., *Randomized, placebo-controlled, double-blind study assessing the efficacy and safety of paliperidone palmitate in Asian patients with schizophrenia.* Neuropsychiatric Disease and Treatment, 2013: p. 1889.

108.	van Kammen, D.P., et al., *A randomized, controlled, dose-ranging trial of sertindole in patients with schizophrenia.* Psychopharmacology, 1996. **124**(1-2): p. 168-175.

109.	DeMartinis, N., et al., *Poster #212 Results of a phase 2A proof-of-concept trial with a PDE10A inhibitor in the treatment of acute exacerbation of schizophrenia Schizophrenia Research*, 2012. **136**: p. S262.

110.	Zborowski, J., et al., *Efficacy and safety of sertindole in a trial of schizophrenic patients.* Biological Psychiatry, 1995. **37**(9): p. 661-662.

111.	Zimbroff, DL.*,* et al.*: Controlled, dose response study of sertindole and haloperidol in the treatment of schizophrenia.* Am J Psychiatry,1997.**154**:p.782-791.

### Table S3: Summary characteristics of studies with usable data

| Characteristic | Value |
| --- | --- |
| N studies with usable data | 112 |
| N participants used | 37096 |
| N antipsychotic drugs used (different applications) | 16(26) |
| baseline median positive syndrome schizophrenia scores (IQR, range) | 24.42(22.63 to 25.8, 7.67 to 30.2) |
| baseline median negative syndrome schizophrenia scores (IQR, range) | 23.42(21.79 to 24.66, 16.82 to 27.22) |
| N studies in subgroups | Adult chronic participants with an acute exacerbation: Positive symptoms 95, Negative symptoms 96; Participants with predominant negative symptoms: 4; First episode: 3; Children or Adolescents: 8; Older adults: 0 |
| Median study duration (IQR, range) | 6(6 to 8, 3 to 26) weeks |
| Median age of participants (IQR, range) | 38.6(36.45to 40.55, 14.7to 46.2) years |
| Median duration of illness (IQR, range) | 13.93(11.07to 16.12, 0.3 to 24.8) years |
| Median percentage of women (IQR, range) | 30.4(23.99 to 38.48, 0 to 63.61) % |
| (Grand) Mean percentage of women in the dataset | 32.34% |

N=number, IQR= Inter-quartile range

# Appendix 8: Risk of bias assessment

## Assessments of the risk of bias of individual studies used in the meta-analysis

### Table S4: Risk of bias for individual studies assessed by Cochrane risk of bias tool 1^1^

| **Study name** | **Random sequence generation** | **Allocation concealment** | **Blinding of participants and personnel** | **Blinding of outcome assessment** | **Incomplete outcome** | **Selective reporting** | **Other bias** | **Overall** |
| --- | --- | --- | --- | --- | --- | --- | --- | --- |
| Arvanitis 1997 | Low risk | Unclear risk | Low risk | Low risk | Low risk | Low risk | Low risk | Low risk |
| Beasley 1996a | Low risk | Low risk | Low risk | Low risk | Low risk | Low risk | Low risk | Low risk |
| Beasley 1996b | Low risk | Low risk | Low risk | Low risk | Low risk | Low risk | Low risk | Low risk |
| Beasley 1997 | Low risk | Low risk | Low risk | Low risk | Low risk | Low risk | Low risk | Low risk |
| Berger 2008 | Low risk | Low risk | Low risk | Unclear risk | Low risk | Low risk | Low risk | Low risk |
| Boyer 1995 | Low risk | Low risk | Low risk | Low risk | High risk | High risk | Low risk | Low risk |
| Bugarski-Kirola 2014 | Unclear risk | Unclear risk | Unclear risk | Unclear risk | Low risk | Low risk | Low risk | Unclear risk |
| Cantillon 2014 | Unclear risk | Unclear risk | Unclear risk | Unclear risk | Unclear risk | Unclear risk | Low risk | Unclear risk |
| Canuso 2010b | Low risk | Low risk | Low risk | Low risk | Low risk | Low risk | Low risk | Low risk |
| Casey 2008 | Unclear risk | Unclear risk | Unclear risk | Unclear risk | Low risk | High risk | Low risk | Unclear risk |
| Chouinard 1993 | Low risk | Low risk | Low risk | Low risk | Low risk | Low risk | Low risk | Low risk |
| Citrome 2021 | Low risk | Low risk | Low risk | Low risk | Low risk | High risk | Low risk | Low risk |
| Cooper 2000a | Low risk | Low risk | Low risk | Low risk | Low risk | Low risk | Low risk | Low risk |
| Correll 2015 | Low risk | Unclear risk | Unclear risk | Unclear risk | Low risk | Low risk | Low risk | Low risk |
| Correll 2020 | Low risk | Low risk | Low risk | Low risk | Low risk | Low risk | Low risk | Low risk |
| Correll 2020b | Low risk | Low risk | Low risk | Low risk | Low risk | Low risk | Low risk | Low risk |
| Corrigan 2004 | Low risk | Low risk | Low risk | Low risk | Low risk | High risk | Low risk | Low risk |
| Cutler 2006 | Unclear risk | Unclear risk | Low risk | Low risk | Low risk | High risk | Low risk | Low risk |
| Cutler 2008 | Low risk | Low risk | Low risk | Low risk | High risk | Low risk | Low risk | Low risk |
| Cutler 2008a | Unclear risk | Unclear risk | Low risk | Low risk | Low risk | Low risk | Low risk | Low risk |
| Daniel 1999 | Low risk | Unclear risk | Unclear risk | Unclear risk | Low risk | Low risk | Low risk | Low risk |
| Danion 1999 | Low risk | Low risk | Low risk | Low risk | Low risk | Low risk | Low risk | Low risk |
| Davidson 2007 | Low risk | Low risk | Low risk | Low risk | Low risk | Low risk | Low risk | Low risk |
| Durgam 2014 | Unclear risk | Unclear risk | Unclear risk | Unclear risk | Low risk | Low risk | Low risk | Unclear risk |
| Durgam 2015 | Unclear risk | Unclear risk | Unclear risk | Unclear risk | Low risk | Low risk | Low risk | Unclear risk |
| Durgam 2016a | Unclear risk | Unclear risk | Unclear risk | Unclear risk | Low risk | Unclear risk | Low risk | Unclear risk |
| Egan 2013 | Low risk | Low risk | Low risk | Low risk | Low risk | Low risk | Low risk | Low risk |
| Fabre 1995 | Unclear risk | Unclear risk | Unclear risk | Unclear risk | Low risk | Low risk | Low risk | Unclear risk |
| Findling 2008 | Low risk | Low risk | Low risk | Low risk | Low risk | High risk | Low risk | Low risk |
| Findling 2012 | Low risk | Unclear risk | Low risk | Low risk | Low risk | Low risk | Low risk | Low risk |
| Findling 2015 | Low risk | Low risk | Low risk | Low risk | Low risk | Low risk | Low risk | Low risk |
| Garcia 2009 | Low risk | Unclear risk | Low risk | Low risk | Low risk | Low risk | Low risk | Low risk |
| Goff 1998 | Unclear risk | Unclear risk | Unclear risk | Unclear risk | Low risk | Low risk | Low risk | Unclear risk |
| Goff 2013 | Unclear risk | Unclear risk | Low risk | Low risk | Unclear risk | Low risk | Low risk | Low risk |
| Goldman 2017 | Low risk | Low risk | Low risk | Low risk | Low risk | Low risk | Low risk | Low risk |
| Gopal 2010 | Low risk | Low risk | Low risk | Low risk | Low risk | Low risk | Low risk | Low risk |
| Hale 2000 | Low risk | Low risk | Low risk | Low risk | Low risk | Low risk | Low risk | Low risk |
| Hass 2009 | Low risk | Unclear risk | Unclear risk | Unclear risk | Low risk | Low risk | Low risk | Low risk |
| Higuchi 2019a | Low risk | Unclear risk | Low risk | Low risk | Low risk | Low risk | Low risk | Low risk |
| Higuchi 2019b | Unclear risk | Unclear risk | High risk | High risk | Low risk | Low risk | Low risk | Low risk |
| Hirayasu 2010 | Unclear risk | Unclear risk | Low risk | Low risk | Unclear risk | Unclear risk | Low risk | Unclear risk |
| Honer 2010 | Low risk | Low risk | Unclear risk | Unclear risk | Low risk | Low risk | Low risk | Low risk |
| Ishigooka 2018 | Low risk | Low risk | Unclear risk | Unclear risk | Low risk | Low risk | Low risk | Low risk |
| Iwata 2019 | Low risk | Low risk | Low risk | Low risk | Low risk | Low risk | Low risk | Low risk |
| Iyo 2021 | Low risk | Low risk | Low risk | Low risk | Low risk | Low risk | Low risk | Low risk |
| Kahn 2007 | Unclear risk | Unclear risk | Low risk | Low risk | Low risk | Low risk | Low risk | Low risk |
| Kane 2002 | Unclear risk | Unclear risk | Unclear risk | Unclear risk | Low risk | Low risk | Low risk | Unclear risk |
| Kane 2003 | Low risk | Low risk | Low risk | Low risk | Low risk | Low risk | Low risk | Low risk |
| Kane 2007b | Low risk | Low risk | Low risk | Low risk | Low risk | Low risk | Low risk | Low risk |
| Kane 2010a | Unclear risk | Unclear risk | Low risk | Low risk | Unclear risk | Low risk | Low risk | Low risk |
| Kane 2014 | Low risk | Low risk | Low risk | Low risk | Low risk | Low risk | Low risk | Low risk |
| Kane 2015a | Low risk | Low risk | Low risk | Low risk | Low risk | Low risk | Low risk | Low risk |
| Kane 2015b | Unclear risk | Unclear risk | Unclear risk | Unclear risk | Low risk | Low risk | Low risk | Unclear risk |
| Keck 1998 | Low risk | Unclear risk | Unclear risk | Unclear risk | Low risk | Low risk | Low risk | Low risk |
| King 1998 | Low risk | Low risk | Unclear risk | Unclear risk | Low risk | Low risk | Low risk | Low risk |
| Kinon 2006c | Low risk | Low risk | Low risk | Low risk | Low risk | Low risk | Low risk | Low risk |
| Kinon 2011 | Low risk | Low risk | Low risk | Low risk | High risk | Low risk | Low risk | Low risk |
| Kinoshita 2016 | Low risk | Low risk | Low risk | Low risk | Low risk | Low risk | Low risk | Low risk |
| Kramer 2010 | Low risk | Low risk | High risk | High risk | Low risk | Low risk | Low risk | Low risk |
| Landbloom 2016 | Low risk | Unclear risk | Low risk | Low risk | Low risk | Low risk | Low risk | Low risk |
| Lauriello 2008 | Low risk | Low risk | Unclear risk | Unclear risk | Low risk | Low risk | Low risk | Low risk |
| Lecrubier 2006 | Low risk | Low risk | Low risk | Low risk | Low risk | High risk | Low risk | Low risk |
| Liebermann 2015 | Low risk | Low risk | Low risk | Low risk | Low risk | Low risk | Low risk | Low risk |
| Lindenmayer 2008 | Low risk | Low risk | Low risk | Low risk | Low risk | Low risk | Low risk | Low risk |
| Lindenmayer 2011 | Unclear risk | Unclear risk | Low risk | Low risk | Low risk | High risk | Low risk | Low risk |
| Litman 2016 | Low risk | Unclear risk | Low risk | Low risk | Low risk | Low risk | Low risk | Low risk |
| Litmann 2014 | Low risk | Low risk | Low risk | Low risk | Low risk | Low risk | Low risk | Low risk |
| Loebel 2013 | Low risk | Low risk | Low risk | Low risk | Low risk | Low risk | Low risk | Low risk |
| Loo 1997 | Low risk | Low risk | Low risk | Low risk | Low risk | High risk | High risk | Low risk |
| Marder 1994 | Low risk | Low risk | Low risk | Low risk | Low risk | Low risk | Low risk | Low risk |
| Marder 2007c | Low risk | Low risk | Low risk | Low risk | Low risk | Low risk | Low risk | Low risk |
| Matsumoto 2018 | Low risk | Low risk | Unclear risk | Unclear risk | Low risk | Low risk | Low risk | Low risk |
| McEvoy 2007b | Low risk | Low risk | Low risk | Low risk | Low risk | Low risk | Low risk | Low risk |
| Meltzer 2004 | Low risk | Unclear risk | Unclear risk | Unclear risk | Low risk | Low risk | Low risk | Low risk |
| Meltzer 2007a | Unclear risk | Unclear risk | Unclear risk | Unclear risk | Low risk | Unclear risk | Unclear risk | Unclear risk |
| Meltzer 2011 | Low risk | Low risk | Low risk | Low risk | Low risk | Low risk | Low risk | Low risk |
| Meltzer 2012 | Unclear risk | Unclear risk | Unclear risk | Unclear risk | Low risk | Low risk | Low risk | Unclear risk |
| Meltzer 2014 | Unclear risk | Unclear risk | Unclear risk | Unclear risk | Unclear risk | High risk | Low risk | Unclear risk |
| Meltzer 2015 | Unclear risk | Unclear risk | Low risk | Low risk | Low risk | Low risk | Low risk | Low risk |
| Meltzer 2020 | Unclear risk | Unclear risk | Low risk | Low risk | Low risk | Low risk | Low risk | Low risk |
| Merlo 2000 | Unclear risk | Unclear risk | Unclear risk | Unclear risk | Unclear risk | Unclear risk | Unclear risk | Unclear risk |
| Nakamura 2009 | Low risk | Low risk | Low risk | Low risk | Low risk | Low risk | Low risk | Low risk |
| Nakamura 2016 | Unclear risk | Unclear risk | High risk | High risk | Low risk | Low risk | Low risk | Low risk |
| Nasrallah 2010 | Low risk | Low risk | Low risk | Low risk | Low risk | Low risk | Low risk | Low risk |
| Nasrallah 2013 | Low risk | Low risk | Low risk | Low risk | Low risk | Low risk | Low risk | Low risk |
| Nasser 2016 | Low risk | Low risk | Low risk | Low risk | Low risk | Low risk | Low risk | Low risk |
| NCT00905307 | Low risk | Low risk | Low risk | Low risk | Low risk | Low risk | Low risk | Low risk |
| Ogasa 2012 | Low risk | Low risk | Low risk | Low risk | Low risk | Low risk | Low risk | Low risk |
| Oosthuizen 2004 | Unclear risk | Unclear risk | Low risk | Low risk | Low risk | Low risk | Low risk | Low risk |
| Pandina 2010 | Low risk | Low risk | Low risk | Low risk | Unclear risk | Unclear risk | Low risk | Low risk |
| Patil 2007 | Unclear risk | Unclear risk | Unclear risk | Unclear risk | Low risk | High risk | Low risk | Unclear risk |
| Peuskens 1995 | Low risk | Low risk | Low risk | Low risk | Low risk | Low risk | Low risk | Low risk |
| Potkin 2003 | Unclear risk | Unclear risk | Unclear risk | Unclear risk | Low risk | High risk | Low risk | Unclear risk |
| Potkin 2007c | Unclear risk | Unclear risk | Low risk | Low risk | Low risk | Low risk | Low risk | Low risk |
| Potkin 2008a | Low risk | Low risk | Low risk | Low risk | Low risk | Low risk | Low risk | Low risk |
| Potkin 2008b | Low risk | Low risk | Low risk | Low risk | Low risk | Low risk | Low risk | Low risk |
| Potkin 2008c | Low risk | Low risk | Low risk | Low risk | Low risk | Low risk | Low risk | Low risk |
| Potkin 2015 | Low risk | Low risk | Low risk | Low risk | Low risk | Low risk | Low risk | Low risk |
| Puech 1998 | Low risk | Low risk | Low risk | Low risk | Low risk | Low risk | Low risk | Low risk |
| Saito 2020 | Low risk | Low risk | Low risk | Low risk | Low risk | Low risk | Low risk | Low risk |
| Schmidt 2014 | Low risk | Low risk | Low risk | Low risk | Low risk | High risk | Low risk | Low risk |
| Shen 2014 | Low risk | Low risk | Unclear risk | Unclear risk | High risk | High risk | Low risk | Low risk |
| Singh 2011 | Low risk | Low risk | Low risk | Low risk | Unclear risk | Low risk | Low risk | Low risk |
| Study 115 2000 | Low risk | Unclear risk | Unclear risk | Unclear risk | Low risk | Unclear risk | Low risk | Unclear risk |
| Study 93202 2002 | Unclear risk | Unclear risk | Unclear risk | Unclear risk | Low risk | Low risk | Low risk | Unclear risk |
| Study 94202 2002 | Unclear risk | Unclear risk | Low risk | Low risk | Low risk | Low risk | Low risk | Low risk |
| Study RIS-USA-72 1996 | Unclear risk | Unclear risk | Low risk | Low risk | Low risk | Low risk | Low risk | Low risk |
| Takahashi 2013 | Unclear risk | Unclear risk | Low risk | Low risk | Low risk | Low risk | Low risk | Low risk |
| van Kammen 1996 | Low risk | Low risk | Unclear risk | Unclear risk | Low risk | Low risk | Low risk | Low risk |
| Walling 2019 | Low risk | Low risk | Low risk | Low risk | Low risk | Low risk | Low risk | Low risk |
| Zborowski 1995 | Low risk | Low risk | Low risk | Low risk | Low risk | Low risk | Low risk | Low risk |
| Zimbroff 1997 | Low risk | Low risk | Low risk | Low risk | Low risk | Low risk | Low risk | Low risk |

**References**

1. Higgins JPT, Green S. *Cochrane Handbook for Systematic Reviews of Interventions Version 5.1.0 [updated March 2011].* Chichester, UK: Wiley and Sons; 2011.

## Summary of risk of bias of included studies used in the meta-analysis

### Figure S1: Summary of risk of bias of included studies assessed by Cochrane risk of bias tool 1

# Appendix 9: Summary of the statistical results of the meta-analysis

### Table S5: Statistical summary of the participants with acute exacerbation

| **Drug** | **N (studies)** | **N (participants, positive/ negative)** | **Wald test (df=2)** | | **Coefficient 1** | | **Coefficient 2** | |
| --- | --- | --- | --- | --- | --- | --- | --- | --- |
|  |  |  | **X^2^** | **p-value** | **Mean (95% CI)** | **p-value** | **Mean (95% CI)** | **p-value** |
| Amisulpride | 1 | 248 | 7.2849 | 0.0262 | -0.0012 (-0.0020 to -0.0003) | 0.0076 | 0.0012 (0.0002 to 0.0022) | 0.0185 |
|  |  |  | 1.1767 | 0.5552 | -0.0005 (-0.0013 to 0.0004) | 0.3004 | 0.0006 (-0.0005 to 0.0016) | 0.2788 |
| Aripiprazole | 11 | 3083 | 30.1496 | 0.0000 | -0.0473 (-0.0676 to -0.0270) | 0.0000 | 0.0233 (0.0101 to 0.0364) | 0.0005 |
|  |  | 3098 | 85.4276 | 0.0000 | -0.0464 (-0.0594 to -0.0334) | 0.0000 | 0.0242 (0.0143 to 0.0340) | 0.0000 |
| Asenapine | 5 | 1782 | 42.6955 | 0.0000 | -0.0503 (-0.0669 to -0.0338) | 0.0000 | 0.0405 (0.0200 to 0.0610) | 0.0001 |
|  |  | 1781 | 17.2949 | 0.0002 | -0.0385 (-0.0567 to -0.0204) | 0.0000 | 0.0320 (0.0107 to 0.0533) | 0.0032 |
| Blonanserin | 2 | 817 | 20.7433 | 0.0000 | -0.0732(-0.1905 to 0.0442) | 0.2216 | 0.0348 (-0.0669 to 0.1365) | 0.5025 |
|  |  |  | 14.3893 | 0.0008 | -0.0788 (-0.1646 to 0.0069) | 0.0714 | 0.0404 (-0.0304 to 0.1112) | 0.2632 |
| Brexpiprazole | 4 | 2131 | 8.9106 | 0.0116 | -0.0946 (-0.2229 to 0.0336) | 0.1481 | 0.0653 (-0.0965to 0.2271) | 0.4288 |
|  |  | 2136 | 26.9703 | 0.0000 | -0.2049 (-0.3112 to -0.0985) | 0.0002 | 0.2024 (0.0598 to 0.3451) | 0.0054 |
| Cariprazine | 5 | 1881 | 37.7612 | 0.0000 | 0.0975 (-0.1390 to -0.0560) | 0.0000 | 0.0293 (0.0087 to 0.1236) | 0.0241 |
|  |  |  | 20.1264 | 0.0000 | 0.0009 (-0.1747 to -0.0448) | 0.0009 | 0.1022 (0.0184 to 0.1861) | 0.0169 |
| Haloperidol | 15 | 2362 | 129.3952 | 0.0000 | -0.0752(-0.1044 to -0.0461) | 0.0000 | 0.0450 (0.0141 to 0.0847) | 0.0061 |
|  |  | 2360 | 41.9549 | 0.0000 | -0.0582 (-0.0905 to -0.0259) | 0.0004 | 0.0473 (0.0053 to 0.0775) | 0.0172 |
| Iloperidone | 4 | 1849 | 30.3048 | 0.0000 | -0.0287 (-0.0530 to -0.0044) | 0.0205 | 0.2856 (-0.0110 to 0.0372) | 0.2856 |
|  |  |  | 15.1085 | 0.0005 | -0.0147(-0.0390 to 0.0095) | 0.2343 | 0.0034(-0.0206 to 0.0275) | 0.7795 |
| Lumateperone | 2 | 668 | 12.7136 | 0.0017 | -0.0161 (-0.0286 to -0.0036) | 0.0117 | 0.0080 (-0.0015 to 0.0175) | 0.0979 |
|  |  |  | 4.3371 | 0.1143 | -0.0084(-0.0164 to -0.0004) | 0.0401 | 0.0058 (-0.0014 to 0.0130) | 0.1146 |
| Lurasidone | 10 | 3182 | 46.2447 | 0.0000 | -0.0078 (-0.0108 to -0.0049) | 0.0000 | 0.0043 (0.0017 to 0.0070) | 0.0012 |
|  |  |  | 21.0699 | 0.0000 | -0.0065 (-0.0100 to -0.0034) | 0.0000 | 0.0041 (0.0014 to 0.0068) | 0.0029 |
| Olanzapine | 19 | 4075 | 150.0579 | 0.0000 | -0.0626 (-0.0744 to -0.0508) | 0.0000 | 0.0295 (0.0208 to 0.0382) | 0.0000 |
|  |  |  | 83.5482 | 0.0000 | -0.0489 (-0.0744 to -0.0508) | 0.0000 | 0.0190 (0.0015 to 0.0365) | 0.0326 |
| Paliperidone | 10 | 3905 | 86.6605 | 0.0000 | -0.0987 (-0.0987 to -0.0700) | 0.0000 | 0.0393 (0.0204 to 0.0582) | 0.0000 |
|  |  |  | 59.9441 | 0.0000 | -0.0836 (-0.1083 to -0.0589) | 0.0000 | 0.0396(0.0223 to 0.0569) | 0.0000 |
| Quetiapine | 8 | 2363 | 9.5626 | 0.0084 | -0.0010 (-0.0016 to -0.0003) | 0.0030 | 0.0005 (0.0001 to 0.0009) | 0.0231 |
|  | 9 | 2980 | 10.4247 | 0.0054 | -0.0006 (-0.0011 to -0.0001) | 0.0134 | 0.0002 (-0.0002 to 0.0006) | 0.2527 |
| Risperidone | 20 | 5446 | 267.2162 | 0.0000 | -0.1826 (-0.2080 to -0.1579) | 0.0000 | 0.0919 (0.0758 to 0.1080) | 0.0000 |
|  |  |  | 89.1221 | 0.0000 | -0.1232 (-0.1553 to -0.0911) | 0.0000 | 0.0693 (0.0440 to 0.0946) | 0.0000 |
| Sertindole | 4 | 1273 | 24.7274 | 0.0000 | -0.0209 (-0.0361 to -0.0058) | 0.0069 | 0.0050 (-0.0126 to 0.0225) | 0.5785 |
|  |  | 1270 | 17.7008 | 0.0001 | -0.0177(-0.0400 to 0.0046) | 0.1202 | 0.0035(-0.0263 to 0.0332) | 0.8201 |
| Ziprasidone | 6 | 1152 | 27.4371 | 0.0000 | -0.0041 (-0.0071 to -0.0011) | 0.0081 | 0.0021 (-0.0028 to 0.0070) | 0.4051 |
|  | 5 | 1079 | 20.3219 | 0.0000 | -0.0037(-0.0059 to -0.0014) | 0.0016 | 0.0021(-0.0002 to 0.0045) | 0.0789 |

N = number; df = degrees of freedom; CI = confidence interval. For each drug, the first row shows data for positive symptoms, and the second for negative symptoms. If the values are the same, only one cell is provided. Coefficients 1 and 2 are explained in the general note below.

### Table S6: Statistical summary of the participants with predominant negative symptoms

| **Drug** | **N (studies)** | **N (participants, positive/negative)** | **Wald test (df=2)** | | **Coefficient 1** | | **Coefficient 2** | |
| --- | --- | --- | --- | --- | --- | --- | --- | --- |
|  |  |  | **X^2^** | **p-value** | **Mean (95% CI)** | **p-value** | **Mean (95% CI)** | **p-value** |
| Amisulpride | 4 | 589 | 2.9502 | 0.2288 | -0.0012(-0.0020 to -0.0003) | 0.0076 | 0.0012(0.0002 to 0.0022) | 0.0185 |
|  |  | 585 | 30.8785 | 0.0000 | -0.0005(-0.0013 to 0.0004) | 0.3004 | 0.0006(-0.0005 to 0.0016) | 0.2788 |
| Olanzapine | 1 | 174 | 7.181 | 0.0276 | -0.1185 (-0.2059 to -0.0311) | 0.0079 | 0.2052 (0.0547 to 0. 0.3557) | 0.0075 |
|  |  |  | 3.1945 | 0.2024 | -0.0554(-0.1421 to 0.0312) | 0.2100 | 0.1135(-0.0359 to 0.2629) | 0.1366 |

N = number; df = degrees of freedom; CI = confidence interval. For each drug, the first row shows data for positive symptoms, and the second for negative symptoms. If the values are the same, only one cell is provided. Coefficients 1 and 2 are explained in the general note below.

### Table S7: Statistical summary of the children or adolescent participants

| **Drug** | **N (studies)** | **N (participants, positive/ negative)** | **Wald test (df=2)** | | **Coefficient 1** | | **Coefficient 2** | |
| --- | --- | --- | --- | --- | --- | --- | --- | --- |
|  |  |  | **X^2^** | **p-value** | **Mean (95% CI)** | **p-value** | **Mean (95% CI)** | **p-value** |
| Aripiprazole | 2 | 399 | 8.9501 | 0.0114 | -0.0311 (-0.0615 to -0.0008) | 0.0442 | 0.0332 (-0.0166 to 0.0831) | 0.1909 |
|  |  |  | 1.8366 | 0.3992 | 0.0015(-0.0756 to 0.0787) | 0.9686 | -0.0127(-0.1446 to 0.1192) | 0.8505 |
| Asenapine | 1 | 228 | 10.8945 | 0.0043 | -0.0675 (-0.1420 to 0.0070) | 0.0756 | 0.0192 (-0.0702 to 0.1086) | 0.6739 |
|  |  |  | 4.1063 | 0.1283 | -0.0600(-0.1343 to 0.0145) | 0.1143 | 0.0408(-0.0487 to 0.1302) | 0.3718 |
| Blonanserin | 1 | 150 | 7.4232 | 0.0244 | -0.0402 (-0.0974 to 0.0170) | 0.1683 | 0.0074 (-0.0602 to 0.0750) | 0.8302 |
|  |  |  | 5.9416 | 0.0513 | -0.0278(-0.0850 to 0.0293) | 0.3402 | -0.0041(-0.0717 to 0.0635) | 0.9050 |
| Lurasidone | 1 | 326 | 25.5719 | 0.0000 | -0.0165 (-0.0243 to -0.0088) | 0.0000 | 0.0120(0.0028 to 0.0213) | 0.0110 |
|  |  |  | 7.3143 | 0.0258 | -0.0093(-0.0170 to -0.0016) | 0.0172 | 0.0075(-0.0018 to 0.0167) | 0.1124 |
| Paliperidone | 1 | 200 | 7.5683 | 0.0562 | -0.1292 (-0.2607 to 0.0023) | 0.0542 | 0.1153 (-0.0386 to 0.2692) | 0.1420 |
|  |  |  | 3.8782 | 0.1438 | -0.1317(-0.2633 to -0.0002) | 0.0500 | 0.1453(-0.0089 to 0.2994) | 0.0647 |
| Quetiapine | 1 | 220 | 7.5683 | 0.0227 | -0.0009 (-0.0018 to 0.0001) | 0.0749 | 0.0004 (-0.0007 to 0.0015) | 0.4872 |
|  |  |  | 1.8400 | 0.3985 | -0.0006(-0.0015 to 0.0004) | 0.2333 | 0.0005(-0.0007 to 0.0016) | 0.4265 |
| Risperidone | 1 | 255 | / | / | / | / | / | / |

/: Cannot be analyzed due to lack of data

N = number; df = degrees of freedom; CI = confidence interval. For each drug, the first row shows data for positive symptoms, and the second for negative symptoms. If the values are the same, only one cell is provided. Coefficients 1 and 2 are explained in the general note below.

General note for Appendix 9:
The dose-response relationship was modeled using a restricted cubic spline (RCS) with knots at the 25^th^, 50^th^, and 75^th^ percentiles of the dose distribution, except for asenapine, for which knots were placed at the 10^th^, 50^th^, and 90^th^ percentiles. The RCS with three knots is parameterized by two coefficients: coefficient 1 represents the overall linear component, and coefficient 2 captures the potential non-linear deviation from linearity^1^.

**References**

1. Crippa, A., and N. Orsini, Dose-response meta-analysis of differences in means. *BMC Medical Research Methodology*, 2016. 16: p. 91.

# Appendix 10: Dose-Dependent Effects of Antipsychotics on Positive and Negative Symptoms in different patients groups (with CIs)

Red is for positive symptoms, black for negative symptoms.

### Figure S2 Positive/negative symptoms change of individual antipsychotics in different dosages in adult participants with acute exacerbations of chronic schizophrenia

### Figure S3 Positive/negative symptoms change of individual antipsychotics in different dosages in participants with predominant negative symptoms of chronic schizophrenia

### Figure S4 Positive/negative symptoms change of individual antipsychotics in different dosages in child/ adolescent participants with acute exacerbations of chronic schizophrenia

# Appendix 11: Sensitivity analyses

### Figure S5 Dose-Dependent Effects of Antipsychotics on Positive and Negative Symptoms: Exclusion of Single-Dose vs. Placebo Comparisons

Red solid lines represent the main analyses for positive symptoms, black ones for negative symptoms, dotted lines showing sensitivity analyses separately. Amisulpride for participants with predominant negative symptoms, aripiprazole, asenapine, haloperidol, iloperidone, lurasidone, olanzapine, paliperidone, quetiapine, risperidone, and ziprasidone for participants with chronic conditions and acute exacerbations are included (adults only).

### Figure S6 Dose-Dependent Effects of Antipsychotics on Positive and Negative Symptoms: Exclusion of studies in treatment-resistant patients

Red solid lines represent the main analyses for positive symptoms, black ones for negative symptoms, dotted lines showing sensitivity analyses separately. Lurasidone, quetiapine, risperidone, and ziprasidone for participants with chronic conditions and acute exacerbations are included (adults only).

### Figure S7 Dose-Dependent Effects of Antipsychotics on Positive and Negative Symptoms: Separate analyses of different formulations of antipsychotics.

LAI: long-acting injectable antipsychotic, IR: immediate release analyses, XR: extended-release analyses. Aripiprazole, asenapine, blonanserin, olanzapine, paliperidone, quetiapine and risperidone for participants with chronic conditions and acute exacerbations are included (adults only).

### Figure S8 Olanzapine-equivalent dose–response curves across antipsychotics excluding haloperidol.

Red solid lines represent the main analyses for positive symptoms, black ones for negative symptoms, dotted lines showing sensitivity analyses separately.

# Appendix 12: Heterogeneity assessments

## Heterogeneity assessments with the variance-partition-coefficient (VPC) for participants with acute exacerbation

1. Amisulpride: Only one study was included, thus no heterogeneity was assessed.
2. Aripiprazole:
   1) Aripiprazole for positive symptoms: Median VPC across doses was around 40%, indicating moderate level of heterogeneity.
   2) Aripiprazole for negative symptoms: Median VPC across doses was 0%, indicating no heterogeneity.
3. Asenapine:
   1) Asenapine for positive symptoms: Median VPC across doses was around 15%, indicating low level of heterogeneity.
   2) Asenapine for negative symptoms: Median VPC across doses was around 40%, indicating moderate level of heterogeneity.
4. Blonanserin：
   1) Blonanserin for positive symptoms: There was considerable level of heterogeneity with median VPC across doses up to 60%.
   2) Blonanserin for negative symptoms: Median VPC across doses was around 25%, indicating moderate level of heterogeneity.
5. Brexpiprazole:
   1) Brexpiprazole for positive symptoms: Median VPC across doses was around 5%, indicating low level of heterogeneity.
   2) Brexpiprazole for negative symptoms: Median VPC across doses was 0%, indicating no heterogeneity.
6. Cariprazine:
   1) Cariprazine for positive symptoms: Median VPC across doses was under 5%, indicating low level of heterogeneity.
   2) Cariprazine for negative symptoms: Median VPC across doses was around 30%, indicating moderate level of heterogeneity.
7. Haloperidol:
   1) Haloperidol for positive symptoms: Median VPC across doses was under 5%, indicating low level of heterogeneity.
   2) Haloperidol for negative symptoms: Median VPC across doses was under 20%, indicating low level of heterogeneity.
8. Iloperidone:
   1) Iloperidone for positive symptoms: Median VPC across doses was 0%, indicating no heterogeneity.
   2) Iloperidone for negative symptoms: Median VPC across doses was 0%, indicating no heterogeneity.
9. Lumateperone:
   1) Lumateperone for positive symptoms: There was considerable level of heterogeneity with median VPC across doses up to 60%.
   2) Lumateperone for negative symptoms: There was considerable level of heterogeneity with median VPC across doses up to 50%.
10. Lurasidone:
    1) Lurasidone for positive symptoms: Median VPC across doses was around 25%, indicating moderate level of heterogeneity.
    2) Lurasidone for negative symptoms: Median VPC across doses was around 25%, indicating moderate level of heterogeneity.
11. Olanzapine:
    1) Olanzapine for positive symptoms: Median VPC across doses was under 20%, indicating low level of heterogeneity.
    2) Olanzapine for negative symptoms: Median VPC across doses was under 40%, indicating moderate level of heterogeneity.
12. Paliperidone:
    1) Paliperidone for positive symptoms: Median VPC across doses was around 30%, indicating moderate level of heterogeneity.
    2) Paliperidone for negative symptoms: Median VPC across doses was around 25%, indicating moderate level of heterogeneity.
13. Quetiapine:
    1) Quetiapine for positive symptoms: There was considerable level of heterogeneity with median VPC across doses up to 80%.
    2) Quetiapine for negative symptoms: There was considerable level of heterogeneity with median VPC across doses up to 60%.
14. Risperidone:
    1) Risperidone for positive symptoms: Median VPC across doses was around 10%, indicating low level of heterogeneity.
    2) Risperidone for negative symptoms: Median VPC across doses was around 25%, indicating moderate level of heterogeneity.
15. Sertindole:
    1) Sertindole for positive symptoms: Median VPC across doses was under 5%, indicating low level of heterogeneity.
    2) Sertindole for negative symptoms: Median VPC across doses was around 25%, indicating moderate level of heterogeneity.
16. Ziprasidone:
    1) Ziprasidone for positive symptoms: Median VPC across doses was under 10%, indicating low level of heterogeneity.
    2) Ziprasidone for negative symptoms: Median VPC across doses was 0%, indicating no heterogeneity.

## VPC for participants with predominant negative symptoms

1. Amisulpride:
   1) Amisulpride for positive symptoms: There was considerable level of heterogeneity with median VPC across doses up to 60%.
   2) Amisulpride for negative symptoms: Median VPC across doses was under 20%, indicating low level of heterogeneity.
2. Olanzapine: Only one study was included, thus no heterogeneity was assessed.

## VPC for child/adolescent participants

1. Aripiprazole:
   1) Aripiprazole for positive symptoms: Median VPC across doses was 0%, indicating no heterogeneity.
   2) Aripiprazole for negative symptoms: There was considerable level of heterogeneity with median VPC across doses up to 60%.
2. Asenapine: Only one study was included, thus no heterogeneity was assessed.
3. Blonanserin: Only one study was included, thus no heterogeneity was assessed.
4. Lurasidone: Only one study was included, thus no heterogeneity was assessed.
5. Paliperidone: Only one study was included, thus no heterogeneity was assessed.
6. Quetiapine: Only one study was included, thus no heterogeneity was assessed.
7. Risperidone: Only one study was included, thus no heterogeneity was assessed.

# Appendix 13: Small-study effect and publication bias assessments

We have conducted pairwise meta-analyses comparing antipsychotic groups (all doses combined) with placebo groups in aripiprazole (11 studies), haloperidol (15 studies), olanzapine (17 studies), paliperidone (10 studies), and risperidone (17 studies).

Then, funnel plots for each aforementioned drug were conducted for assessments. We detected no publication bias for these studies since Egger’s test for small study effects bias was not significant and the funnel plot was symmetrical, except for positive symptoms of haloperidol (p=0.0088).

SD: Standard Deviation, SMD: Standardized Mean Difference, CI: Confidence Interval.

Forest and funnel plots are presented as below. A for positive symptoms and B for negative symptoms.

### Figure S9: Forest plot of Aripiprazole (all doses combined) compared to placebo

A

B

### Figure S10: Forest plot of Haloperidol (all doses combined) compared to placebo

A

B

### Figure S11: Forest plot of Olanzapine (all doses combined) compared to placebo

A

B

### Figure S12: Forest plot of Paliperidone (all doses combined) compared to placebo

A

B

### Figure S13: Forest plot of Risperidone (all doses combined) compared to placebo

A

B

### Figure S14: Funnel plot for Aripiprazole studies

A

B

### Figure S15: Funnel plot for Haloperidol studies

A

B

### Figure S16: Funnel plot for Olanzapine studies

**A**

**B**

### Figure S17: Funnel plot for Paliperidone studies

A

B

### Figure S18: Funnel plot for Risperidone studies

A

B
